# The two enantiomers of 2-hydroxyglutarate differentially regulate cytotoxic T cell function

**DOI:** 10.1016/j.celrep.2023.113013

**Published:** 2023-08-24

**Authors:** Iosifina P. Foskolou, Pedro P. Cunha, Elena Sánchez-López, Eleanor A. Minogue, Benoît P. Nicolet, Auré lie Guislain, Christian Jorgensen, Sarantos Kostidis, Nordin D. Zandhuis, Laura Barbieri, David Bargiela, Demitris Nathanael, Petros A. Tyrakis, Asis Palazon, Martin Giera, Monika C. Wolkers, Randall S. Johnson

**Affiliations:** 1 Department of Physiology, Development and Neuroscience, University of Cambridge, Downing Site, Cambridge CB2 3EG, UK; 2 Department of Cell and Molecular Biology (CMB), Karolinska Institutet, Solnavägen 9, 171 65 Solna, Sweden; 3 Department of Hematopoiesis, Sanquin Research and Landsteiner Laboratory Amsterdam University Medical Center, University of Amsterdam, 1066 CX Amsterdam, the Netherlands; 4 Oncode Institute, 3521 AL Utrecht, the Netherlands; 5 Leiden University Medical Center, Center for Proteomics and Metabolomics, Albinusdreef 2, 2333ZA Leiden, the Netherlands; 6 Department of Chemistry, Aarhus University, Langelandsgade 140, 8000 Aarhus C, Denmark

## Abstract

2-Hydroxyglutarate (2HG) is a byproduct of the tricarboxylic acid (TCA) cycle and is readily detected in the tissues of healthy individuals. 2HG is found in two enantiomeric forms: S-2HG and R-2HG. Here, we investigate the differential roles of these two enantiomers in cluster of differentiation (CD)8^+^ T cell biology, where we find they have highly divergent effects on proliferation, differentiation, and T cell function. We show here an analysis of structural determinants that likely underlie these differential effects on specific α-ketoglutarate (αKG)-dependent enzymes. Treatment of CD8^+^ T cells with exogenous S-2HG, but not R-2HG, increased CD8^+^ T cell fitness *in vivo* and enhanced anti-tumor activity. These data show that S-2HG and R-2HG should be considered as two distinct and important actors in the regulation of T cell function.

## Graphical abstract

**Graphical abstract:**
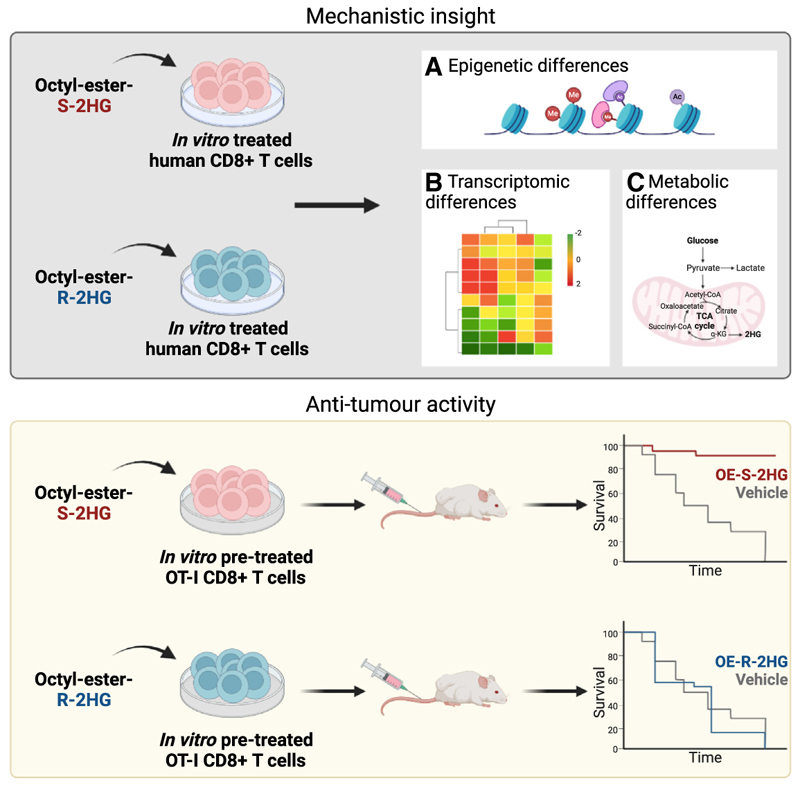

## Introduction

There are four known metabolites with structural similarities to α-ketoglutarate (αKG) that inhibit a range of αKG-dependent enzymes. Two of these, succinate and fumarate, are tricarboxylic acid (TCA) cycle metabolites with essential metabolic roles; the third was recently revealed to be glutarate,^1^ a product of amino acid catabolism. The fourth metabolite is 2-hydroxyglutarate (2HG), a physiological byproduct of the TCA cycle. 2HG has two enantiomers: the S-form (also known as L-2HG) and the R-form (also known as D-2HG). Intracellular accumulation of 2HG is derived by the reduction of αKG to either R-2HG or S-2HG.^2^ Both enantiomers can be detected in most body fluids of healthy individuals, and their concentrations can reach near millimolar levels in urine and serum.^3,4^

R-2HG is the most studied 2HG enantiomer due to its increased levels in tumors harboring mutations in isocitrate dehydrogenase (IDH1/2mut).^2,5–7^ R-2HG can also be physiologically formed by wild-type IDH enzymes and the promiscuous activity of 3-phosphoglycerate dehydrogenase (PHGDH).^8,9^ S-2HG accumulates in hypoxic and acidic conditions through the action of malate dehydrogenase 1 and 2 (MDH1 and MDH2) and lactate dehydrogenase A (LDHA).^9–11^ S-2HG accumulation has also been reported in clear cell renal cell carcinomas (ccRCCs) and pancreatic cancers.^12,13^ However, the role of 2HG in tumorigenesis is more complex than initially thought. For example, patients with IDHmut glioma or acute myeloid leukemia (AML) tend to have better overall survival rates than patients with IDH wild-type (IDH-WT) gliomas or AML.^6,7,14–16^

2HG accumulation is observed in both physiological and pathological conditions, and it is important to determine whether the two 2HG enantiomers act in a similar fashion in immune cells. As both enantiomers can affect cytotoxic T cell differentiation, proliferation, and function,^2,11,17–19^ we wished to determine their different roles in cluster of differentiation (CD)8^+^ T cell functions. We show that the two enantiomers differentially regulate T cell function.

## Results

### Human CD8^+^ T cells express different surface markers when treated with cell-permeable S-2HG or R-2HG

**Figure 1:**
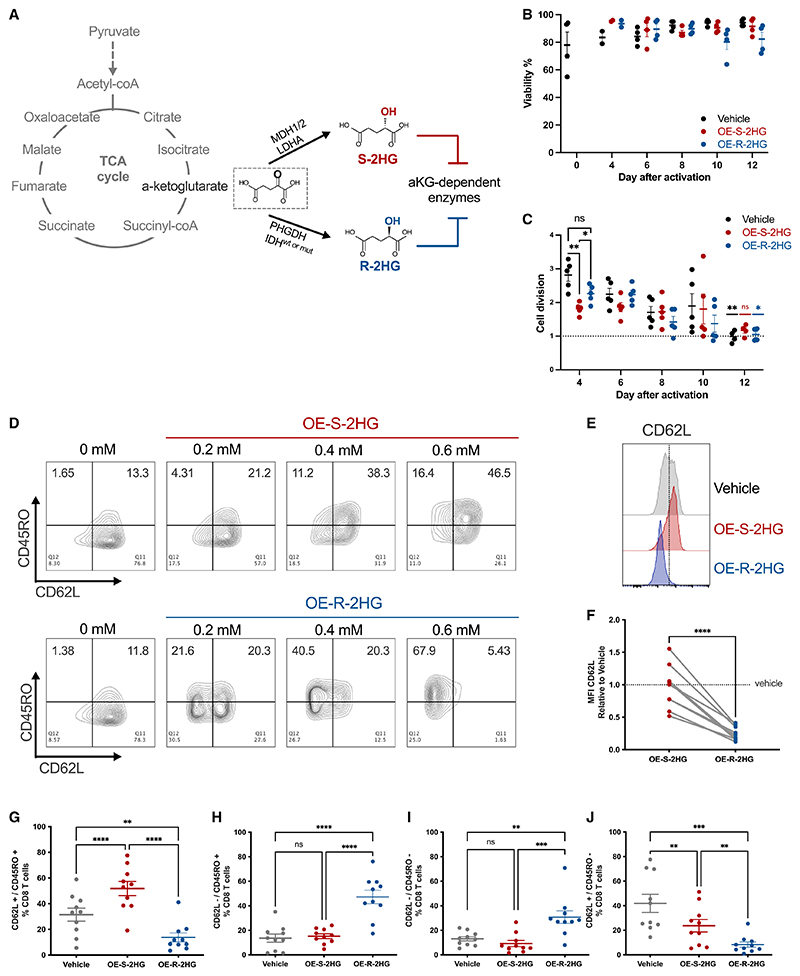
Human CD8^+^ T cells express different surface markers when treated with OE-S-2HG or OE-R-2HG (A) Schematic representation of the TCA cycle and chemical structures of α-ketoglutarate (αKG), S-2-hydroxyglutarate (S-2HG) and R-2-hydroxyglutarate (R-2HG). (B) Viability of CD8^+^ T cells as determined by automated cell counter. Data are represented as mean ± SEM. (C) Cell division of CD8^+^ T cells for the indicated days. Mixed-effects analysis with Tukey’s multiple comparisons test was used (one test comparing treatment at different time points and one test comparing effect cell division at day 12 with day 4 for each treatment). Data are represented as mean ± SEM. (D) Flow cytometry plots of CD8^+^ T cells showing surface expression of CD62L and CD45RO. Representative plots of n = 3 are shown. (E) Histograms of representative flow cytometry plots for CD62L expression on CD8^+^ T cells. (F) Fold change of median fluorescence intensity (MFI) of CD62L for CD8^+^ T cells analyzed by flow cytometry. Each data point represents a donor (n = 9; 6 independent experiments). Unpaired two-tailed Student’s t test was used. (G–J) Frequency of (G) CD62L+/CD45RO+, (H) CD62L–/CD45RO+, (I) CD62L–/CD45RO-, and (J) CD62L+/CD45RO–cells is shown (%CD8^+^ T cells). Each data point represents a donor (n = 10; 7 independent experiments). Data are represented as mean ± SEM. Repeated measures (RM) one-way ANOVA with Tukey’s multiple comparisons test was used. For all panels, naive CD8^+^ T cells were isolated, activated, and treated every 1–2 days with OE-S-2HG (0.4 mM), OE-R-2HG (0.4 mM), or vehicle (H_2_O). Analysis was performed on day 12 unless otherwise stated. For all panels, *p ≤ 0.05; **p ≤ 0.01; ***p ≤ 0.001; ****p ≤ 0.0001. See also Figure S1.

The structural similarity of both 2HG enantiomers to αKG causes them to act as competitive inhibitors of αKG-dependent enzymes (Figure 1A).^20,21^ To investigate the differential effects of the two enantiomers of 2HG on human T cells, we used cell-permeable octyl ester forms of S-2HG (OE-S-2HG) and R-2HG (OE-R-2HG). To minimize donor-to-donor variation, we isolated naive CD8^+^ T (T_N_) cells from peripheral blood mononuclear cells (PBMCs) of healthy individuals, activated them with aCD3/aCD28, and treated them with the two OE-2HG enantiomers every 1–2 days. Neither OE-S-2HG nor OE-R-2HG had an effect on CD8^+^ T cell viability when used in physiologically relevant concentrations (0.4 mM)^3,11^ for up to 12 days of culture (Figure 1B).

Early after activation, CD8^+^ T cells treated with OE-S-2HG proliferated less (Figure S1A) and had a slower rate of cell division (day 4; Figure 1C) when compared with CD8^+^ T cells treated with OE-R-2HG or with vehicle. However, cell division of OE-S-2HG-treated CD8^+^ T cells remained stable over later time points when compared to day 4, whereas cell division of vehicle- and OE-R-2HG-treated CD8^+^ T cells was gradually and significantly decreased (Figure 1C). On day 12, OE-S-2HG-treated CD8^+^ T cells had a cell division rate of 1.169 ± 0.17, followed by OE-R-2HG (1.049 ± 0.19) and vehicle (0.9879 ± 0.18) (Figure 1C). In sum, OE-S-2HG-treated CD8^+^ T cells proliferate slower after activation, but their proliferation is less decreased at later time points relative to OE-R-2HG- and vehicle-treated cells.

We then examined the effect of both enantiomers on the expression of several surface markers related to human CD8^+^ T cell differentiation. Increased concentrations of OE-S-2HG led to a dose-dependent increase of CD62L+/CD45RO+ (Figure 1D) and CCR7+/CD45RO+ (Figure S1B) CD8^+^ T cells, which are markers associated with early differentiated memory-like CD8^+^ T cells. Although increasing concentrations of OE-R-2HG increased the CCR7+/CD45RO+ population (Figure S1B), they led to a dose-ependent loss of CD62L and an increase in CD62L−/CD45RO+ cells (Figures 1D–1F). The differential expression of CD62L and CCR7 determines the differential migratory tendencies of CD8^+^ T cells. T cells expressing both CD62L and CCR7 can recirculate between lymphoid tissues and peripheral blood,^22^ whereas cells that express CCR7 but are CD62L− are usually T cells exiting peripheral tissues and are found in afferent lymphatics.^23,24^

At 0.4 mM, both OE-S-2HG and OE-R-2HG significantly increased the CCR7+/CD45RO+ population compared with vehicle (Figure S1C), but only OE-S-2HG increased the CD62L+/CD45RO+ population compared with both vehicle and OE-R-2HG (Figure 1G). The OE-R-2HG enantiomer significantly increased the CD62L−/CD45RO+ and CD62L−/ CD45RO− populations, and decreased the CD62L+/CD45RO–populations, relative to treatment with either vehicle or OE-S-2HG (Figures 1H–1J). Neither OE-S-2HG nor OE-R-2HG had an effect on CCR7−/CD45RO+ and CCR7−/CD45RO–populations, and only OE-S-2HG-treated populations had decreased CCR7+/CD45RO–CD8^+^ T cells when compared with vehicle-treated cells (Figures S1D–S1F).

Interestingly, OE-R-2HG-treated CD8^+^ T cells increased expression of the homing marker CCR7 but decreased expression of the co-stimulatory marker CD28 relative to CD8^+^ T cells treated with OE-S-2HG (Figures S1G and S1H). OE-R-2HG-treated cells showed a moderate but not significant increase of the activation/checkpoint marker PD-1 and the transcription factor TOX (Figures S1I and S1J). In summary, CD8^+^ T cells treated with the two cell-permeable forms of 2HG show significant differences in cell division, proliferation, and expression of surface markers.

### Transcriptome analysis reveals distinct differences between OE-S-2HG- and OE-R-2HG-treated human CD8^+^ T cells

**Figure 2:**
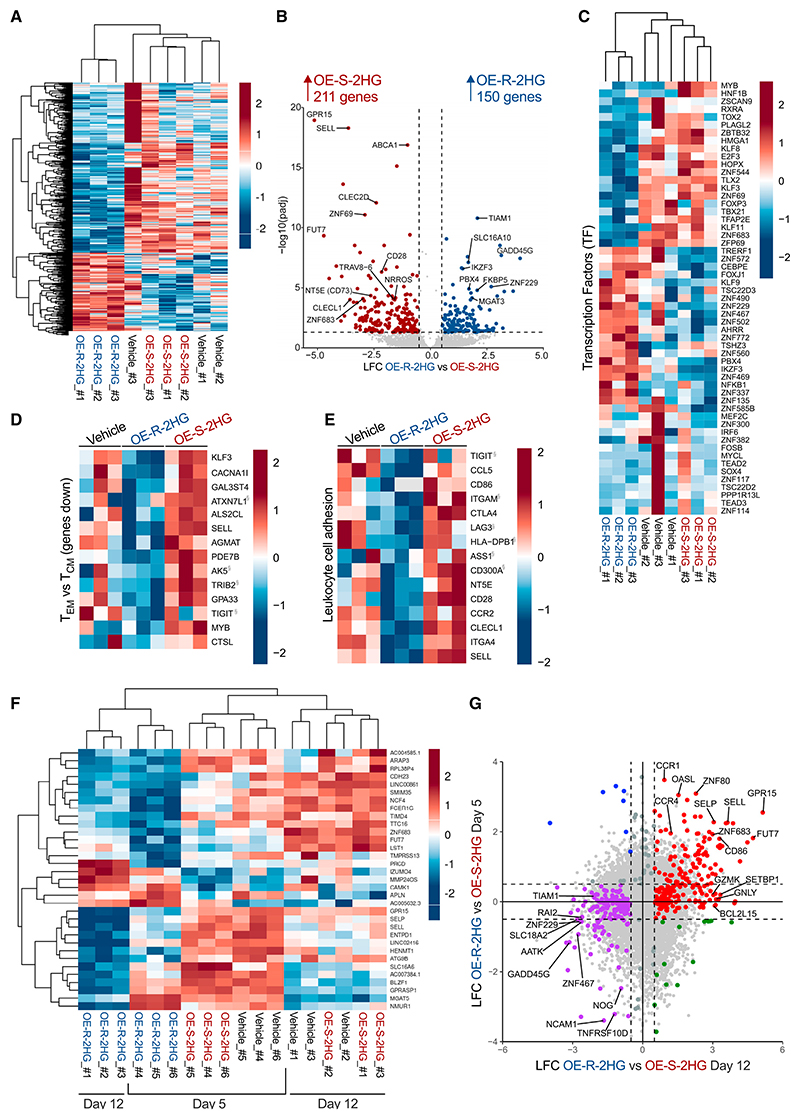
OE-S-2HG- and OE-R-2HG-treated human CD8^+^ T cells have distinct transcriptomes Naive CD8^+^ T cells were isolated from 6 donors over 3 independent experiments, activated, and treated with OE-S-2HG (0.4 mM), OE-R-2HG (0.4 mM), or vehicle (H_2_O). The cells were collected either on day 5 (3 donors) or on day 12 (3 donors), and RNA-seq analysis followed. (A) Heatmap of hierarchically clustered genes at day 12 of culture. (B) Volcano plot showing log_2_ fold change (LFC; x axis) and −log_10_ adjusted p value (y axis) of transcripts differentially expressed in OE-R-2HG-treated CD8^+^ T cells vs. OE-S-2HG-treated CD8^+^ T cells (day 12). Colored dots represent LFC >0.5 (blue) or <−0.5 (red) and adjusted p value [p.adj] <0.05. (C) Heatmap of hierarchically clustered genes in CD8^+^ T cells (day 12). Statistically significant differentially expressed hits of transcription factors are shown. (D and E) Heatmaps of standardized gene expression (*Z* score) in treated CD8^+^ T cells (day 12): (D) downregulated genes in T_EM_ cells compared with T_CM_ cells, and (E) genes involved in leukocyte cell adhesion. Gene sets were obtained from ToppGene. Red and blue colors indicate increased and decreased expression, respectively. Genes marked with “§” were non-statistically significant hits. (F) Heatmap of hierarchically clustered genes in CD8^+^ T cells at days 5 and 12 of culture. (G) LFC of OE-R-2HG vs. OE-S-2HG on day 5 compared with on day 12. Dotted lines represent absolute LFC of 0.5. The red dots indicate statistically significant genes for OE-S-2HG upregulated at day 12 (LFC >0.5), which were also either upregulated or unchanged at day 5 (LFC > −0.5). The purple dots indicate statistically significant genes for OE-R-2HG upregulated at day 12 (LFC < −0.5), which were also either upregulated or unchanged at day 5 (LFC <0.5). See also Figure S2 and Table S1.

To assess if the observed differences in cell proliferation and surface markers were due to transcriptome-related changes between OE-S-2HG- and OE-R-2HG-treated CD8^+^ T cells, we performed RNA sequencing (RNA-seq) analysis of CD8^+^ T_N_ cells early (day 5) and late (day 12) after activation. Cells were isolated, activated, and treated every 1–2 days with OE-S-2HG (0.4 mM), OE-R-2HG (0.4 mM), or vehicle (H_2_O). Hierarchical clustering revealed distinct clusters of transcript expression depending on treatment with OE-S-2HG and OE-R-2HG, or vehicle, at both days 5 (Figure S2A) and 12 (Figure 2A). Volcano plot analysis identified a total of 361 differentially expressed genes (log_2_ fold change >0.5; adjusted p value [p.adj] < 0.05) between OE-S-2HG- and OE-R-2HG-treated cells on day 12 of treatment (Figure 2B). Hierarchical clustering of significant genes revealed differential expression of transcription factors in OE-S-2HG- and OE-R-2HG-treated cells (Figure 2C), as well as CD molecules and secreted molecules (Figures S2B and S2C). Interestingly, in concordance with protein measurements (Figures 1E, 1F, and S1H), OE-S-2HG-treated CD8^+^ T cells expressed higher *SELL* (CD62L) and *CD28* transcript levels compared with OE-R-2HG-treated cells (Figure 2B). Furthermore, OE-S-2HG-treated cells showed higher transcript levels of some genes that are preferentially expressed in CD8^+^ naive (T_N_) and/or central memory (T_CM_) T cells, including *SELL* (CD62L), *CD28, GPR15, NT5E* (CD73), *FUT7*, and *ZNF69* (Table S1; Figure 2B).

Using publicly available datasets, we observed that OE-R-2HG decreased the expression of genes whose expression is usually low in effector memory (T_EM_) CD8^+^ T cells when compared with T_CM_ cells, with the opposite being the case for OE-S-2HG-treated cells (Figure 2D). The gene signature of OE-R-2HG-treated cells was not typical of effector/effector memory cells, as markers associated with cytotoxicity and effector functions were lower in OE-R-2HG-treated cells relative to OE-S-2HG- and vehicle-treated cells (e.g., *ZFN683, HOPX, ZBTB32*, and *TBX21*) (Figures 2B and 2C). In addition, OE-R-2HG-treated cells expressed more *IKZF3* compared with OE-S-2HG-treated cells (Figures 2B and 2C); this gene was shown to be a repressor of effector function in CD4^+^ T cells.^25^

OE-S-2HG-treated cells increased expression of genes important for leukocyte cell adhesion (Figure 2E) and T cell activation (Figure S2D). Furthermore, gene set enrichment analysis (GSEA) revealed that OE-S-2HG-treated T cells displayed enrichment of genes associated with early T lymphocytes, cell-cycling genes, and E2F3 targets compared with OE-R-2HG (Figures S2E–S2G). E2F3 is a transcription factor that interacts directly with the retinoblastoma protein (pRB) to regulate the expression of genes involved in the cell cycle.^26^ These results support our observation that OE-S-2HG-treated cells have a moderately higher cell division rate than OE-R-2HG-treated cells at day 12 of treatment (Figure 1C).

Finally, we questioned whether different time points of treatment with the two enantiomers (days 5 and 12) resulted in different gene expression profiles. Hierarchical clustering of the two time points showed close clustering of OE-R-2HG-treated CD8^+^ T cells, indicating a treatment effect (Figure 2F). In addition, the two most separated T cell populations were those treated for 12 days with OE-R-2HG and OE-S-2HG (Figure 2F), indicating that the longer the cells are treated with either of the two 2HG compounds, the greater the divergence in their transcriptomes. This finding was supported by the log_2_ fold change (LFC) analysis of OE-R-2HG vs. OE-S-2HG at day 5 compared with day 12 (Figures 2G, S2H, and S2I). Interestingly, for OE-S-2HG-treated cells, genes such as *SELL, SELP, FUT7, GPR15, ZNF683, CCR1*, and *CCR4* were upregulated at both time points measured (Figure 2G). These data indicate that OE-S-2HG- and OE-R-2HG-treated cells have different transcriptional targets, which can explain some of the observed differences seen in CD8^+^ T cell proliferation and differentiation after treatment with the two compounds.

### S-2HG and R-2HG differentially inhibit specific αKG-dependent enzymes

We next studied the molecular mechanisms underlying the different transcriptional profiles of OE-S-2HG- and OE-R-2HG-treated CD8^+^ T cells. Because of their structural similarities with αKG, both S-2HG and R-2HG can competitively inhibit αKG-dependent enzymes.^20,21^ Some αKG-dependent enzymes are epigenetic modulators, including histone and DNA demethylases.^21^ Therefore, we first assessed the effect of OE-S-2HG and OE-R-2HG treatments on histone modifications in CD8^+^ T cells.

**Figure 3:**
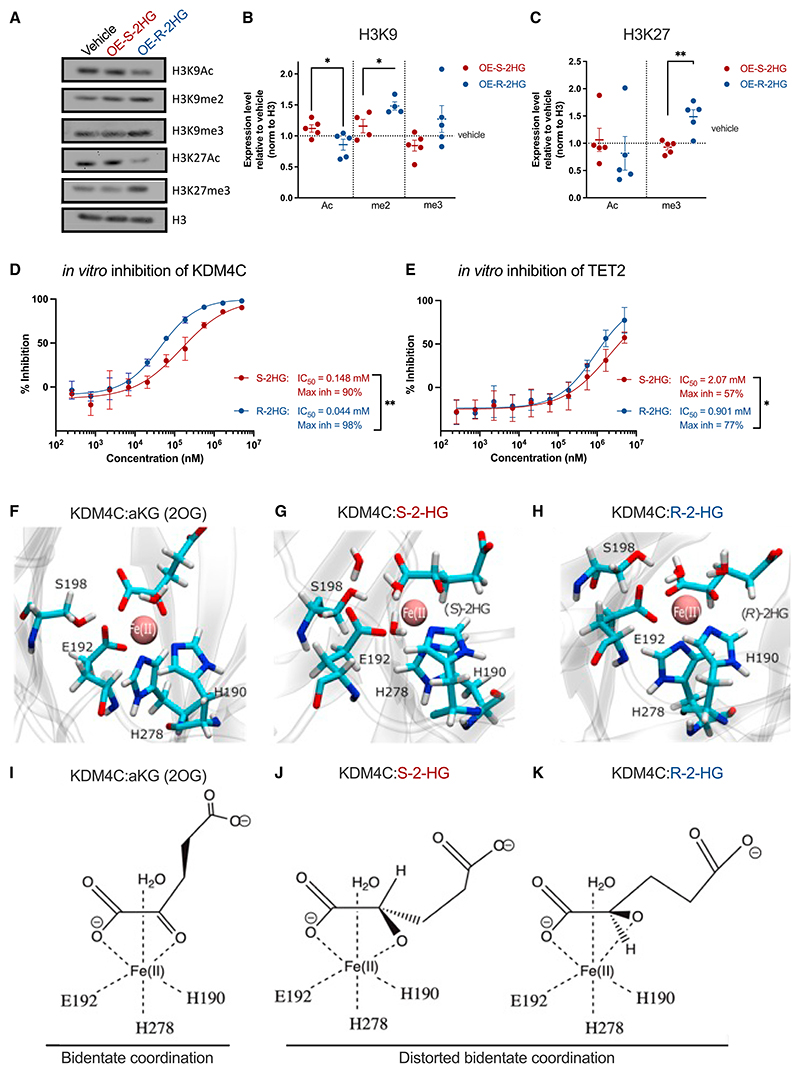
S-2HG and R-2HG have different inhibition potencies for some αKG-dependent enzymes (A) Western blot analysis of naive CD8^+^ T cells activated and treated every 1–2 days with OE-S-2HG (0.4 mM), OE-R-2HG (0.4 mM), or vehicle (H_2_O) for 12 days. Representative images of n = 4 are shown. (B and C) Quantification of the expression levels of different histone marks shown in (A) relative to total H3 for (B) H3K9 and (C) H3K27. Each data point represents a donor (n = 4–5; 4 independent experiments). Data are represented as mean ± SEM. Unpaired two-tailed Student’s t test was used. (D) *In vitro* enzymatic inhibition assay for the KDM4C enzyme. Inhibition was determined by using increasing concentrations of S-2HG or R-2HG. Data are represented as mean ± SD (n = 4; 2 independent experiments). Results are shown as non-linear fit (inhibitor vs. response four parameters variable slope), and two-way ANOVA was used. The statistical analysis shown is concentration × metabolite. *(E) In vitro* enzymatic inhibition assay for the TET2 enzyme. Inhibition was determined by using increasing concentrations of S-2HG or R-2HG. Data are represented as mean ± SD (n = 6; 3 independent experiments). The results are shown as non-linear fit (inhibitor vs. response four parameters variable slope), and two-way ANOVA was used. The statistical analysis shown is concentration × metabolite. (F–H) Representative snapshots of the catalytic site of KDM4C protein (PDB: 4XDO; resolution 1.97 Å) in complex with (F) αKG (KDM4C:2OG:Fe(II)), (G) S-2HG (KDM4C:2HG-(S):Fe(II)), and (H) R-2HG (KDM4C:2HG-(R):Fe(II)). (I) αKG (KDM4C:2OG:Fe(II)) maintains the pocket integrity from the crystal structure and is found in a bidentate coordination. (J) In KDM4C:2HG-(S):Fe(II), the backbone of S-2HG rearranges to maintain a pseudo-bidentate coordination of Fe(II). (K) In KDM4C:2HG-(R):Fe(II), the backbone of R-2HG resembles αKG, and no conformational rearrangement occurs. The enzymatic pockets of KDM4C:2HG-(S):Fe(II) and KDM4C:2HG-(R):Fe(II) contain additional waters near the coordination sphere of Fe(II) as a result of the disruption induced by the change from αKG to S-2HG or R-2HG. For (A), uncropped and ladder images are deposited in Mendeley Data. For all panels, *p ≤ 0.05; **p ≤ 0.01; ***p ≤ 0.001; ****p ≤ 0.0001. See also Figure S3.

OE-S-2HG treatment significantly increased histone 3 lysine 9 acetylation (H3K9ac) compared with OE-R-2HG, whereas OE-R-2HG slightly increased H3K9 tri-methylation (H3K9me3) and significantly increased H3K9 di-methylation (H3K9me2) (Figures 3A and 3B). In addition, OE-R-2HG-treated cells increased H3K27me3 at the expense of H3K27ac compared with OE-S-2HG-treated cells (Figures 3A and 3C). These data are further supported by the broad increase of gene expression of histone deacetylase (HDAC) targets in OE-R-2HG-treated T cells and the decrease in the OE-S-2HG-treated samples (Figure S3A).

We next checked genes that are regulated by enhancer of zeste 2 (EZH2) and SUZ12, two members of the polycomb repressive complex 2 (PRC2), which regulates H3K27me deposition (Figure S3B).^27^ EZH2 is essential for effector CD8^+^ T cell expansion, and PRC2 deficiency impairs effector CD8^+^ T cell differentiation but minimally impacts memory CD8^+^ T cell maturation.^28^ Target genes that are downregulated by SUZ12 were increased in OE-S-2HG-treated T cells and decreased in OE-R-2HG-treated T cells (Figure S3C). Similarly, EZH2-associated genes were significantly increased in OE-R-2HG-treated T cells (Figure S3D). To determine whether S-2HG or R-2HG could functionally alter the activity of histone demethylases, we performed *in vitro* enzymatic activity assays for the H3K9 demethylase KDM4C. In agreement with our cellular data, R-2HG was a more potent inhibitor than S-2HG, and the maximum percentages of KDM4C inhibition with our assay were 98% for R-2HG and 90% for S-2HG (Figure 3D).

We then checked the effect of OE-S-2HG and OE-R-2HG treatments on DNA methylation, which directly dictates CD8^+^ T cell differentiation and function.^29^ The ten-eleven translocation (TET) enzymes are αKG-dependent DNA demethylases that catalyze the oxidation of 5-methylcytocine (5mC) to 5-hydroxymethylcytocine (5hmC).^30,31^ To determine if OE-S-2HG or OE-R-2HG treatment affects DNA methylation, we isolated CD8^+^ T_N_ cells, activated and treated them for 7 days with OE-S-2HG (0.4 mM), OE-R-2HG (0.4 mM), or vehicle (H_2_O), and determined total 5hmC levels by flow cytometry (Figure S3E). Although both OE-S-2HG and OE-R-2HG lowered the 5hmC levels compared with vehicle, no differences were observed between OE-S-2HG and OE-R-2HG treatments in overall 5hmC levels (Figure S3F). We then performed *in vitro* enzymatic activity assays for TET2 using methylated single-stranded DNA (ssDNA) as a substrate. *In vitro*, R-2HG inhibited TET2 slightly more than S-2HG: the maximum inhibition values of TET2 with our assay were 77% for R-2HG and -57% for S-2HG (Figure 3E).

To gain a structural insight into the different inhibitory potencies of the 2HG metabolites, we investigated the conformation of KDM4C (PDB: 4XDO; resolution 1.97 Å) bound with αKG, S-2HG, or R-2HG (Figures 3F–3H and S3G–S3I). Both S-2HG and R-2HG can bind to the active site of the enzyme, albeit in a distorted bidentate coordination when compared with αKG, since the keto carboxyl end of αKG is replaced by a hydroxyl group in S-2HG and R-2HG (Figures 3I–3K). The bidentate coordination of the active site of αKG-dependent enzymes is sensitive to minimal changes,^32^ which explains why both S-2HG and R-2HG can act as inhibitors of KDM4C. R-2HG showed a nearly identical orientation to αKG in the catalytic core of KDM4C, in close proximity to Fe(II) (Figures 3H, 3K, and S3I). In contrast, S-2HG has to twist to find the right conformation in the catalytic core of KDM4C (Figures 3G, 3J, and S3G), which potentially explains why S-2HG is a less effective inhibitor of KDM4C. Collectively, these results highlight that S-2HG and R-2HG can have different affinities for different targets, which may result in different cellular effects.

### OE-S-2HG and OE-R-2HG treatments affect T cell intracellular amino acid and lipid levels

**Figure 4:**
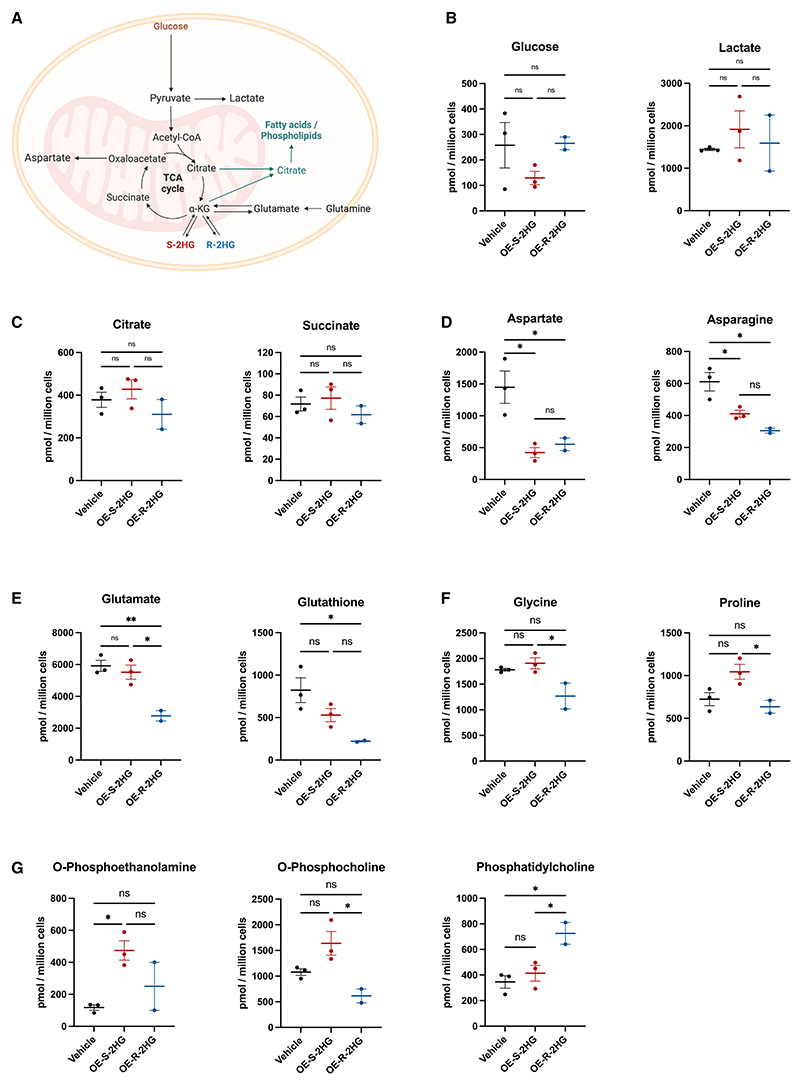
Metabolic changes in OE-S-2HG- and OE-R-2HG-treated naive CD8^+^ T cells (A) Schematic representation of basic intracellular metabolic pathways. (B–G) Naive CD8^+^ T cells were isolated from 4 donors (one of the dots is 2 donors pooled; 2 donors for OE-R-2HG), activated, and treated every 1–2 days with OE-S-2HG (0.4 mM), OE-R-2HG (0.4 mM), or vehicle (H_2_O). The cells were collected on day 7, and NMR-based metabolomics analysis followed. The levels of (B) glucose and lactate; (C) citrate and succinate; (D) aspartate and asparagine; (E) glutamate and glutathione (reduced form); (F) glycine and proline; and (G) O-phosphoethanolamine, O-phosphocholine, and phosphatidylcholine are shown. For all panels, data are represented as mean ± SEM, and ordinary one-way ANOVA with Tukey’s multiple comparisons tests were used. For all panels, *p ≤ 0.05; **p ≤ 0.01; ***p ≤ 0.001; ****p ≤ 0.0001. See also Figure S4.

To investigate the intracellular metabolic roles of S-2HG and R-2HG (Figure 4A), we performed nuclear magnetic resonance (NMR)-based metabolomics on CD8^+^ T cells treated with OE-S-2HG, OE-R-2HG, or vehicle. We isolated human total CD8^+^ and CD8^+^ T_N_ cells and then activated and treated them with OE-S-2HG (0.4 mM), OE-R-2HG (0.4 mM), or vehicle (H_2_O) every 1–2 days for the indicated times (Figures 4 and S4).

For total CD8^+^ T cells, there were no differences between treatments in the levels of glucose and pyruvate either before or after activation (days 4 and 12) (Figures S4A and S4B). At day 4 after activation, lactate levels were significantly decreased in OE-S-2HG-treated compared with vehicle-treated CD8^+^ T cells; at day 12, lactate levels were lower than on day 4 and were similar between treatments (Figure S4C). Early after activation, CD8^+^ T cells utilize glycolysis to boost growth and maintain high proliferation rates. The fact that OE-S-2HG-treated cells had lower lactate levels compared with vehicle at day 4 after activation (Figure S4C) is in agreement with the lower cell division rates observed on the same day (Figures 1C and S1A). We also checked the intracellular levels of 2HG for both OE-S-2HG- and OE-R-2HG-treated CD8^+^ T cells. Surprisingly, we observed higher accumulation of free R-2HG than of S-2HG in the respectively treated CD8^+^ T cells, although the OE forms of 2HG were found intracellularly at similar levels for both OE-S-2HG- and OE-R-2HG-treated cells (Figures S4D and S4E). This result would imply that S-2HG might be more efficiently utilized and/or exported than R-2HG in human CD8^+^ T cells.

We then checked the levels of different metabolites in isolated CD8^+^ T_N_ cells activated and treated with OE-S-2HG (0.4 mM), OE-R-2HG (0.4 mM), or vehicle for 7 days. No differences were observed between treatments in the levels of glucose and lactate at day 7 after activation (Figure 4B). OE-S-2HG- and OE-R-2HG-treated CD8^+^ T cells had similar levels of the TCA metabolites citrate and succinate (Figure 4C). However, OE-S-2HG and OE-R-2HG treatments showed differences in the levels of metabolites closely related to the TCA cycle (Figure 4A). Both OE-S-2HG- and OE-R-2HG-treated cells had lower levels of aspartate and its downstream amino acid asparagine compared with the vehicle-treated cells (Figure 4D). OE-R-2HG-treated cells had lower levels of glutamate and its downstream metabolite glutathione (reduced form) compared with vehicle and OE-S-2HG (for glutamate) treatments (Figure 4E). Aspartate and glutamate are produced in part by enzymes that use αKG in the reactions they catalyze (αKG-dependent transaminases) (Figure S4F). R-2HG can inhibit the αKG-dependent transaminases BCAT1/2 (branched chain amino acid transaminases 1/2) more potently than S-2HG, and R-2HG accumulation lowers glutamate production in glioblastoma cells.^33^ This is in agreement with our observations in CD8^+^ T cells (Figure 4E) and implies that the reduction of glutamate in OE-R-2HG-treated cells could be in part due to the differential inhibition of αKG-dependent transaminases.

We also observed differences in the levels of other amino acids and phospholipids. Specifically, OE-S-2HG-treated cells had higher levels of glycine and proline compared with OE-R-2HG-treated cells (Figure 4F). OE-S-2HG-treated cells had also higher levels of the phospholipid precursors phosphoethanolamine (compared with vehicle-treated cells) and phosphocholine (compared with OE-R-2HG-treated cells), whereas OE-R-2HG-treated cells increased phosphatidylcholine (Figure 4G). Phosphoethanolamine is converted to phosphatidylethanolamine (PE), which together with phosphatidylcholine (PC) comprise most of the mitochondrial membrane lipids.^34^ Interestingly, synthesis of another essential phospholipid for mitochondrial membranes maintains CD8^+^ T cell function, mitochondrial fitness, and memory differentiation.^35^ In sum, OE-S-2HG- and OE-R-2HG-treated cells have distinct differences in central carbon metabolism, which could affect multiple aspects of CD8^+^ T cell function and differentiation.

### OE-S-2HG-treated, but not OE-R-2HG-treated, mouse CD8^+^ T cells show increased tumor infiltration and anti-tumor activity

We next investigated the functional consequences of the observed differences between OE-S-2HG- and OE-R-2HG-treated CD8^+^ T cells in murine adoptive cell transfer (ACT) *in vivo* models. Prior to adoptive T cell transfer, we determined the expression of CD44 (adhesion receptor), CD62L, CD25 (interleukin-2 receptor A), CTLA4 (immune checkpoint receptor), ICOS (T cell co-stimulator), and granzyme B (GzmB) (effector molecule) of the mouse CD8^+^ T cells treated with vehicle, OE-S-2HG, or OE-R-2HG (Figures S5A and S5B). Even though mouse CD8^+^ T cells treated with OE-R-2HG do not lose CD62L expression as their human counterparts do (Figures S5B and 1E), we detected more CD62L+/CD44+ (T_CM_) mouse CD8^+^ T cells when treated with OE-S-2HG compared with treatment with OE-R-2HG or vehicle (Figure S5A).

**Figure 5:**
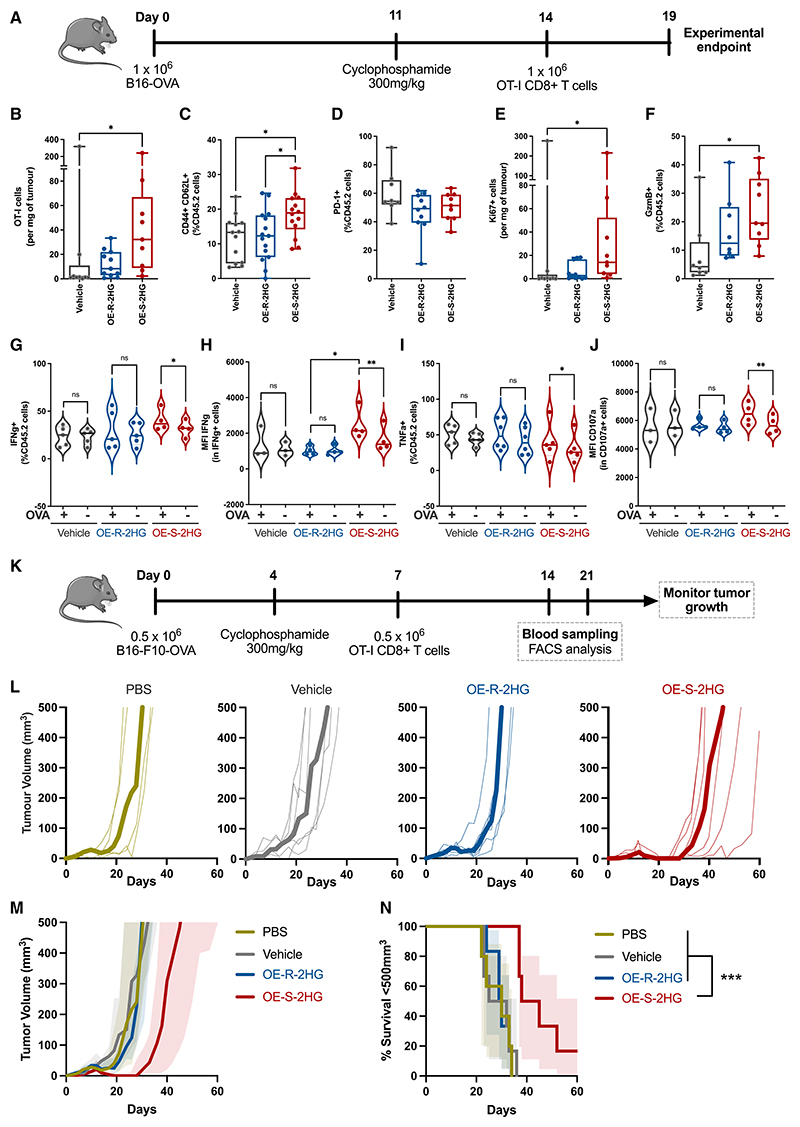
OE-S-2HG-treated mouse CD8^+^ T cells show increased tumor infiltration and anti-tumor activity (A) Schematic representation of the adoptive cell therapy (ACT) *in vivo* model. B16-OVA-tumor-bearing C57BL/6j mice (CD45.1+/CD45.2+) were lymphodepleted with cyclophosphamide and adoptively transferred with CD45.2+ OT-I CD8^+^ T cells, which were previously activated and treated *in vitro* with OE-S-2HG (0.4 mM), OE-R-2HG (0.4 mM), or vehicle (H_2_O) for 7 days. (B) Number of OT-I cells per mg of tumor (n = 9–11 mice per condition, two independent experiments; non-parametric Kruskal-Wallis with Dunn’s multiple comparisons test). (C) Frequency of OT-I cells expressing CD62L+/CD44+ infiltrated in the tumors (n = 14–15 mice per condition, three independent experiments; ordinary one-way ANOVA with Holm-Sidak’s multiple comparisons test). (D) Frequency of OT-I cells expressing PD-1 infiltrated in the tumors (n = 9–10 mice per condition, two independent experiments; ordinary one-way ANOVA with Tukey’s multiple comparisons test). (E) Number of OT-I cells in the tumors positive for Ki67 per mg of tumor (n = 9–11 mice per condition, two independent experiments; non-parametric Kruskal-Wallis with Dunn’s multiple comparisons test). (F) Frequency of OT-I cells infiltrated in the tumors expressing GzmB (n = 8–9 mice per condition, two independent experiments; non-parametric Kruskal-Wallis with Dunn’s multiple comparisons test). (G–J) *In vitro* restimulation of OT-I tumor-infiltrated lymphocytes with OVA_257-264_ (100 nM) peptide. (G) Frequency of OT-I cells expressing IFNγ with (−OVA) or without (-OVA) restimulation. (H) IFNγ MFI of OT-I cells (CD45.2+, IFNγ+) cells with (−OVA) or without (-OVA) restimulation. (I) Frequency of OT-I cells expressing TNF-α with (+OVA) or without (−OVA) restimulation. (J) CD107a MFI of OT-I cells (CD45.2+, CD107a+) cells with (+OVA) or without (−OVA) restimulation. (K) Schematic representation of the ACT *in vivo* model. B16-F10-OVA-bearing C57BL/6j mice were lymphodepleted with cyclophosphamide and adoptively transferred with PBS or CD45.1+ OT-I CD8^+^ T cells, which were activated and treated *in vitro* with OE-S-2HG (0.4 mM), OE-R-2HG (0.4 mM), or vehicle (H_2_O) for 7 days. (L) Tumor growth for each condition. Thin lines represent tumor growth from individual mice, and thick lines represent median tumor sizes for each group. (M) Combined data from (L). (N) Survival curves for tumor growth shown in (L) and (M). Threshold for survival was set at 500 mm^3^. Green line: PBS (n = 5 mice); gray line: vehicle (H_2_O) (n = 6 mice); blue line: OE-R-2HG (n = 6 mice); red line: OE-S-2HG (n = 6 mice). Log-rank (Mantel-Cox) test was used. A similar tumor growth experiment with an EG7-OVA-expressing tumor model is deposited in Mendeley Data. For (B)–(F), median and min to max with all points (individual mice) are shown. For (G)–(J), violin plots with median and all points (individual mice) are shown. Representative of n = 2–3 independent experiments is shown (cumulative data from n = 3 are deposited in Mendeley Data). One-way ANOVA with Tukey’s multiple comparisons was used between treatments, and paired two-tailed Student’s t test was used for each treatment with (+OVA) compared with without (−OVA) restimulation. For (B) and (E), cell number was defined by counting beads. For all panels, *p ≤ 0.05; **p ≤ 0.01; ***p ≤ 0.001; ****p ≤ 0.0001. See also Figure S5.

B16-OVA-bearing C57BL/6j mice treated with cyclophosphamide on day 11 received congenically marked OT-I CD8^+^ T cells at day 14 that were pre-treated with OE-S-2HG, OE-R-2HG, or vehicle (H_2_O) (Figure 5A). Five days later, we analyzed the infiltrated OT-I T cells in the tumors, spleens, and lymph nodes. The overall numbers of OT-I cells in the tumors were higher when T cells were pre-treated with OE-S-2HG compared with vehicle (Figures 5B and S5C). Also, even though similar numbers of OT-I cells were found in spleens (Figure S5D), mice that received OE-S-2HG-treated OT-I cells had significantly bigger spleens than the mice that received vehicle-treated OT-I cells (Figure S5E). Within the draining or non-draining lymph nodes, there were no differences in the numbers of OT-I T cells between treatments (Figure S5F).

Tumor-infiltrating OT-I T cells treated with OE-S-2HG expressed more CD44+/CD62L+ (T_CM_) than OE-R-2HG- and vehicle-treated cells (Figure 5C). The activation marker PD-1 was unaltered in these cells (Figure 5D). We then checked the proliferation potential of the tumor-infiltrated OT-I cells by staining for Ki67. Although the percentage of OT-I cells positive for Ki67 was similar between treatments (Figure S5G), the number of Ki67+ cells was increased in tumors of mice that received OE-S-2HG-treated OT-I cells compared with vehicle-treated cells (Figure 5E). We also found more GzmB-expressing OT-I cells between OE-S-2HG and vehicle, similar to what was seen in our *in vitro* data (Figures 5F, S5A, and S5B).

Tumor-infiltrating lymphocytes (TILs) usually lose the capacity to react to T cell receptor (TCR) activation upon extraction from the tumor.^36^ To investigate whether the treatment with the 2HG enantiomers could reverse this TCR block, we checked effector molecule production with or without *in vitro* restimulation with the cognate OVA_257-264_ peptide. OE-S-2HG-treated OT-I cells significantly increased the percentage and the expression of the cytokine interferon γ (IFNγ) after OVA restimulation (Figures 5G and 5H), which implies that the tumor-infiltrated OT-I cells had decreased anergy. OE-S-2HG-treated OT-I cells also increased the percentage of positive cells, but not the expression levels, of the cytokine tumor necrosis factor α (TNF-α) after restimulation (Figures 5I and S5H). We also checked the degranulation marker CD107a and found increased expression levels, but not the percentage, after restimulation only in OE-S-2HG-treated OT-I cells (Figures 5J and S5I). In contrast, OE-R-2HG- and vehicle-treated OT-I cells did not show increased expression after OVA restimulation for any of the tested markers. The fact that a high percentage of OE-S-2HG-treated OT-I cells were preserved in a T_CM_ (CD44+/CD62L+) phenotype within the tumor might explain the decreased anergy we observed when restimulating these cells *in vitro*.

We then determined the effect of both 2HG enantiomers on CD8^+^ T cell anti-tumor activity by injecting C57BL/6j mice with B16-F10 OVA-expressing tumor cells and adoptively transferring OT-I CD8^+^ T cells that had been pre-treated with OE-S-2HG, OE-R-2HG, or vehicle (Figure 5K). At days 14 and 21 after ACT, we determined the number of OT-I T cells in circulation by flow cytometry (Figure 5K). We found significantly more OE-S-2HG-treated OT-I cells in circulation compared with OE-R-2HG-(day 14) and vehicle-treated (days 14 and 21) OT-I T cells (Figure S5J). Also, the percentage of CD62L+/CD44+ cells was higher for OE-S-2HG-treated OT-I cells compared with vehicle at day 21 (Figure S5K). OE-S-2HG-treated OT-I cells had also increased CD127 (interleukin-7 receptor A) expression compared with vehicle at day 21 (Figure S5L).

Finally, we measured the effect of 2HG enantiomers on T cells relative to their capacity to control tumor growth. OE-S-2HG-treated OT-I cells had superior anti-tumor activity compared with all other conditions tested (OE-R-2HG, vehicle, and PBS, no T cell control), resulting in delayed tumor growth and increased survival (Figures 5L–5N). In contrast, OE-R-2HG OT-I cells had no beneficial effect on anti-tumor activity compared with vehicle-treated OT-I cells (Figure 5L–5N). This result can be explained by the increased number of TILs observed in the tumors (Figure 5B) and that these TILs express more GzmB and can produce high amounts of cytokines after *in vitro* restimulation (Figures 5F–5J). Thus, the differences in gene expression, differentiation status, and function observed in OE-S-2HG-treated CD8^+^ T cells result in improved anti-tumor activity.

## Discussion

Owing to the description of 2HG as an oncometabolite, there are currently many studies of 2HG in T cell immunology and some with contradictory findings. Bunse et al. reported that non-cell-permeable tumor-derived R-2HG can be taken up by human CD8^+^ T cells through the sodium-dependent dicarboxylate transporter 3 (SLC13A3) and that R-2HG accumulation in CD8^+^ T cells suppresses proliferation and anti-tumor activity.^37^ Notarangelo et al. showed that the effect of non-cell-permeable R-2HG on the impairment of CD8^+^ T cells is transient and relies on inhibiting the LDH enzyme.^19^ Conversely, Bottcher et al. failed to detect impaired CD4^+^ or CD8^+^ T cell proliferation or increased cell death when cells were cultured with R-2HG. Rather, they found that R-2HG increased the frequency of regulatory T cells (Tregs).^38^ Other studies concluded that R-2HG accumulation in IDH mutant tumors inhibits both CD8^+^ T cell function and anti-tumor activity.^39–42^

There are also contradictory data for S-2HG, although fewer studies have been conducted. Gupta et al. reported that pancreatic cancers accumulate S-2HG due to the promiscuous activity of LDHA and that LDHA inhibition increased CD8^+^ T cell infiltration. The authors used high concentrations of cell-permeable S-2HG *in vitro*, observed a reduction in CD8^+^ T cell migration, and concluded that S-2HG suppresses anti-tumor activity.^13^ In contrast, we reported that exogenous treatment of mouse and human CD8^+^ T cells with OE-S-2HG favors a memory phenotype and increases anti-tumor activity in multiple adoptive T cell transfer models.^11,17^

Here, we show that treatment of human CD8^+^ T cells with esterified S-2HG or R-2HG differentially modulates CD8^+^ T cell proliferation and differentiation, which is reflected in their differential gene expression of transcription factors, CD molecules, and secreted molecules. OE-S-2HG-treated CD8^+^ T cells expressed more memory-like markers and had a higher proliferative potential, a feature that is key for long-term effective T cell responses *in vivo*. Concurrently, OE-S-2HG-treated cells retained expression of effector molecule transcripts. This was reflected also in our *in vivo* experiments, where pre-treatment with OE-S-2HG increased the amount of functional tumor-infiltrated T cells and showed that these cells had increased cytotoxic potential when rechallenged *in vitro*.

We also note aspects of the molecular mechanisms of the divergent effects of the 2HG enantiomers. S-2HG and R-2HG had different inhibitory potencies for specific aKG-dependent enzymes, and R-2HG was a more potent inhibitor than S-2HG of the KDM4C enzyme. Chowdhury et al. showed that S-2HG inhibits more potently than R-2HG the FIH (factor inhibiting HIF), PHD2 (prolyl hydroxylase domain 2), and ALKBH2 (αKG-dependent dioxygenase homolog 2) enzymes, but, similarly to our results, they found that R-2HG inhibits KDMs with greater potency.^21^ R-2HG and S-2HG can also have different potencies for αKG-dependent transaminases,^33^ which results in differential accumulation of specific metabolites and amino acids. We observed that OE-R-2HG-treated cells had lower glutamate and glutathione levels, whereas OE-S-2HG-treated cells had increased glycine and proline levels. Glutathione is an important non-enzymatic anti-oxidant that balances reactive oxygen species (ROS) intracellular levels, and failure to buffer accumulating ROS can block T cell activation.^43^ Glycine is a building block for glutathione and a contributor to nucleotide synthesis, which maintains T cell proliferation.^44^

OE-S-2HG and OE-R-2HG pre-treatments of adoptively transferred OT-I CD8^+^ T cells showed diverged T cell function in our tumor models. Specifically, OE-S-2HG-treated OT-I cells preferentially migrated to tumor sites and preserved their T_CM_ phenotype both within the tumor and in circulation. OE-S-2HG-treated OT-I cells also expressed more GzmB within tumors and produced ample amounts of effector molecules after *in vitro* restimulation. The above is an important characteristic of functional memory CD8^+^ T cells. The increased functionality of the OE-S-2HG-pre-treated OT-I cells was also demonstrated by their increased anti-tumor activity. Interestingly, although OE-R-2HG-pre-treated OT-I cells did not show a beneficial anti-tumor effect, they did not perform worse than the vehicle-treated cells in our *in vivo* models. This is in agreement with previously published data reporting that R-2HG’s presence is required for CD8^+^ T cell impairment.^19^ However, the effect that OE-S-2HG has on CD8^+^ T cells is not transient and seems to be maintained weeks after treatment.

In conclusion, OE-S-2HG pre-treatment before ACT increases CD8^+^ T cell fitness and enhances anti-tumor activity. Clearly, S-2HG and R-2HG are not merely enantiomeric forms but have distinct functions in CD8^+^ T cell biology.

### Limitations of the study

Some of the observed differences in our study could be attributed to the different intracellular accumulation and/or utilization of the free S-2HG and R-2HG despite the similar intracellular levels of their esterified forms (OE-S-2HG, OE-R-2HG). Also, in the tumor growth/survival experiment, female mice were used. Additional experiments with male mice could increase our research generalizability.

## Star⋆Methods

### Key Resources Table

**Table:** 

REAGENT or RESOURCE	SOURCE	IDENTIFIER
Antibodies		
5-Hydroxymethylcytosine; *In vitro* assay & Flow cytometry	Active Motif	39999; RRID:AB_2566808
CCR7 (human); Flow cytometry	BD Biosciences	3D12; RRID:AB_2033950 & RRID:AB_396765
CD107a (mouse); Flow cytometry	eBiosciences	ID4B;RRID:AB_657536
CD127 (mouse); Flow cytometry	Biolegend	A7R34; RRID:AB_1937252
CD25 (mouse); Flow cytometry	eBiosciences	PC61.5; RRID:AB_10671550
CD28 (human); Flow cytometry	Biolegend	CD28.2; RRID:AB_2936698 & RRID:AB_528785
CD44 (mouse); Flow cytometry	Biolegend	IM7; RRID:AB_830786 & RRID:AB_312962
CD45.1 (mouse); Flow cytometry	Biolegend & BD Biosciences	A20; RRID:AB_313494; RRID:AB_1134170 & RRID:AB_2738523
CD45.2 (mouse); Flow cytometry	Biolegend	104; RRID:AB_893352 & RRID:AB_492871
CD45RO (human); Flow cytometry	Biolegend	UCHL1; RRID:AB_2562143 & RRID:AB_2616917
CD62L (human); Flow cytometry	Biolegend	DREG-56; RRID:AB_528857; RRID:AB_493583; RRID:AB_314463 & RRID:AB_893396
CD62L (mouse); Flow cytometry	Biolegend	MEL-14; RRID:AB_313094 & RRID:AB_313092
CD8a (human); Flow cytometry	Biolegend & BD Biosciences	HIT8a; RRID:AB_528884 & SK1; RRID:AB_2722546
CD8a (mouse); Flow cytometry	BD Biosciences	53–6.7; RRID:AB_2732919
CTLA-4 (mouse); Flow cytometry	Biolegend	UC10-4B9; RRID:AB_313254
Dynabeads human T-activator CD3/CD28	Gibco	11132D; RRID:AB_2943359
FcR Block	MACS Miltenyi	120-000-826; RRID:AB_2943360
GzmB (mouse); Flow cytometry	Biolegend	AD2; RRID:AB_2228785
GzmB (mouse); Flow cytometry	BD Biosciences	GB11; RRID:AB_10561690
H3K27Ac (human); Western Blot	Cell Signaling	8173; RRID:AB_10949503
H3K27me3 (human); Western Blot	Cell Signaling	9733; RRID:AB_2616029
H3K9Ac (human); Western Blot	Cell Signaling	9649; RRID:AB_823528
H3K9me2 (human); Western Blot	Cell Signaling	4658; RRID:AB_10544405
H3K9me3 (human); Western Blot	Cell Signaling	13969; RRID:AB_2798355
Histone3 (H3) (human); Western Blot	Cell Signaling	4499; RRID:AB_10544537
HRP-conjugated secondary antibodies; Western Blot	R&D	HAF008; RRID:AB_357235 & HAF007; RRID:AB_357234
ICOS (mouse); Flow cytometry	Biolegend	C398.4A; RRID:AB_10639735
IFN-γ (mouse); Flow cytometry	Biolegend	XMG1.2; RRID:AB_315401
IL-2 (mouse); Flow cytometry	eBiosciences	JES6-5H4; RRID:AB_469490
Ki67 (mouse); Flow cytometry	eBiosciences	SolA15; RRID:AB_2637480
Ki67 (mouse); Flow cytometry	BD Biosciences	B56; RRID:AB_10611571
Mab anti-histone H3K9 Me2-Eu(K) antibody; *In vitro* assay	Cisbio	61KB2KAE; RRID:AB_2943356
PD-1 (human); Flow cytometry	Biolegend	EH12.2H7; RRID:AB_2563593 & RRID:AB_11124107
PD-1 (mouse); Flow cytometry	BD Biosciences	J43; RRID:AB_2742319
Streptavidin-Alexa Fluor 647; *In vitro* assay	Life Technologies	S21374
TNF-α (mouse); Flow cytometry	Biolegend	MP6-XT22; RRID:AB_10900823
TOX (human); Flow cytometry	Fisher Scientific	TXRX10; RRID:AB_2574265
XL665-conjugated Streptavidin; *In vitro* assay	Cisbio	610SAXLA
Biological samples		
Peripheral blood mononuclear cells (PBMCs) were obtained from healthy donors with consent	Cambridge Bioscience; National Health Service (NHS) Blood and Transplant (NHSBT: Addenbrooke’s Hospital, Cambridge, UK); Sanquin Blood bank (Amsterdam, NL)	N/A
Chemicals, peptides, and recombinant proteins		
4-Pyradine dicarboxylic acid	Sigma	04473
Ammonium iron(II) sulfate hexahydrate	Sigma	F3754
BD CellFIX	BD Biosciences	340181
Bovine liver catalase	Sigma	C40
Brefeldin A	Invitrogen	00-4506-51
BSA	Sigma	B2064
Collagenase	Worthington Biochemical	9001-12-1
CountBright™ Absolute Counting Beads	Life Technologies	C36950
Cyclophosphamide	Sigma	C0768
D-α-Hydroxyglutaric acid disodium salt (R-2HG – *in vitro* assays)	Sigma	H8378
Disodium DL-2-hydroxyglutarate-d3	CDN isotopes	D-7496
DNase I (recombinant)	Roche	09852093103
ECL	Sigma	GERPN2106
EDTA	Ambion	AM9260G
EDTA	Sigma	0369
Europium Protein A	Cisbio	61PRAKLB
Ficoll-Paque PLUS density gradient separation	GE Healthcare	71-7167-00 AF
H3K9(Me3)- GGK(Biotin), peptide	Anaspec	AS-64360-1
HEPES	Gibco	15630-080
HTRF Detection Buffer	Cisbio	61DB9RDF
Human IL-2 (recombinant)	Roche	11 011 456 001
Human IL-2 (recombinant) (Proleukin, Aldesleukin)	Clinigen	N/A
Human KDM4C recombinant peptide	BPS Bioscience	50105
Human TET2 recombinant peptide	BPS Bioscience	50162
L-Ascorbic acid	Sigma	A5960
L-DATAN	Sigma	358924
L-α-Hydroxyglutaric acid disodium salt (S-2HG – *in vitro* assays)	Sigma	90790
LC-MS-grade acetic acid ≥99%	Sigma	A6283
LC-MS-grade acetonitrile	Honeywell	34967
LC-MS-grade methanol	Supelco	1.06035.2500
LC-MS-grade water	Honeywell	14263
Live/Dead Fixable Viability Dyes	Invitrogen	L34963 & L10119
Monensin	eBioscience	00-4505-51
N-Oxalylglycine	Sigma	O9390
OneComp eBeads	Invitrogen	01-1111-42
OVA_257–264_ peptide	GenScript	RP10611
Pluronic F-127	PromoCell	PK-CA707-59005
R-2-HG octyl ester Na salt C_13_H_23_NaO_5_. ((2R)-2-Hydroxyglutaric Acid Octyl Ester Sodium Salt) CAS ID: 1391068-16-8	Toronto Research Chemicals	H942595
S-2-HG octyl ester Na salt C_13_H_23_NaO_5_ ((2S)-2-Hydroxyglutaric Acid Octyl Ester Sodium Salt) CAS ID: 1391067-96-1	Toronto Research Chemicals	H942596
SIINFEKL	ProImmune	P093-0A-G
TCEP	Shanghai Yuanye Bio-Technology Company	S16054
Trimethylsilyl propionic-d4-sodium salt (TSP-d4)	Cambridge Isotope Laboratories, Inc.	DLM-48-PK
α-ketoglutarate (α-KG)	Sigma	K3752
β-mercaptoethanol	Thermo Fisher	21985023
Critical commercial assays		
BCA protein assay kit	Abcam	ab207003
CD8^+^ T cell beads (mouse)	MACS Miltenyi	130-104-075
Cytofix/Cytoperm kit Fixation/Permeabilization Kit	BD Biosciences	554714
Foxp3/Transcription Factor Staining Buffer Set	eBioscience	00-5523-00
Histone Extraction Kit	Abcam	ab113476
Naive CD8^+^ T cell isolation kit (human)	MACS Miltenyi	130-093-244
Total CD8^+^T cell isolation kit (human)	MACS Miltenyi	130-096-495
True-Nuclear Transcription Factor Buffer Set	Biolegend	424401
Deposited data		
Mendeley Data	Elsevier inc.	https://doi.org/10.17632/gj337xjxk6.1.
Gene Expression Omnibus (GEO)	NCBI	GSE212738
Experimental models: Cell lines		
Mouse B16-F10-OVA cells (used in the Karolinska Institute)	Velica et al. 4^45^	N/A
Mouse B16-OVA cells (used in Sanquin)	de Witte et al. 4^46^	N/A
Mouse OVA-expressing MEC.B7.SigOVA cells	van Stipdonk et al. 4^47^	N/A
Experimental models: Organisms/strains		
Mouse: B6.SJL-Ptprca Pepcb/BoyJ (Ly5.1)	The Jackson Laboratory	JAX:002014
Mouse: C57BL/6-Tg(TcraTcrb)1100Mjb/J (OT-I)	The Jackson Laboratory	JAX:003831
Mouse: C57BL/6J	Charles River	632
Mouse: C57BL/6J/Ly5.1/Ly5.2	Popovic et al. 4^48^	N/A
Mouse: C57BL/6JRj (Ly5.2)	Janvier Labs	https://www.janvier-labs.com/en/fiche_produit/c57bl-6jrj_mouse/
Recombinant DNA		
ssDNA (ssBiotin 26nt HydMe-C Oligo- Sandard) 5’-/5Biosg/CAGTAGTCTGGACACAC/i5HydMe-dC/GGTCATGA-3’	Genscript	Custom synthesis
ssDNA (ssBiotin 26nt Me-C Oligo - Substrate) /5Biosg/CAGTAGTCTGGACACAC/iMe-dC/GGTCATGA-3’	Genscript	Custom synthesis
Software and algorithms		
CHARMM 36 FF	Brooks et al. 4^49^	N/A
CHARMM-GUI	Lehigh University, Bethlehem	https://www.charmm-gui.org
Conjugate-Gradient minimization	Fletcher and Reeves 5^50^	N/A
FlowJo v10	BD Biosciences	https://www.flowjo.com/
GraphPad Prism v9.5.1	GraphPad Software	https://www.graphpad.com/
Langevin thermostat	Lemons and Gythiel 5^51^	N/A
Molecular Dynamics	Karplus and Petsko; Warshel 5^52^,^53^	N/A
Multi time step Verlet-I/r-RESPA	Tuckerman et al. 5^54^	N/A
NAMD 2.9	Phillips et al. 5^55^	N/A
Nose-Hoover Langevin piston	Nose 5^56^,^57^	N/A
Particle Mesh Ewald (PME)	Darden et al. 5^58^	N/A
SETTLE algorithm	Miyamoto and Kollman 5^59^	N/A
Other		
DMEM	Thermo Scientific	11995065
Fetal Bovine Serum (FBS)	Sigma	F7524
G418 sulfate	Thermo Scientific	10131027
Penicillin and Streptomycin	Sigma	P0781
RPMI media	Gibco	52400-025

## Resource Availability

### Lead contact

Further information and requests for resources and reagents should be directed to and will be fulfilled by the lead contact, Randall S Johnson (rsj33@cam.ac.uk).

### Materials availability

This study did not generate new unique reagents.

## Experimental Model and Study Participant Details

### Mice

C57BL/6J/Ly5.2, C57BL/6J/Ly5.1/Ly5.2 mice and C57BL/6J were bred in-house at the Netherlands Cancer Institute (NKI) or were purchased from Janvier Labs (for animal experiments performed in the Karolinska Institute, Sweden). Donor T cell receptor (TCR) transgenic mice (OT-I) mice were either crossed with mice bearing the CD45.1 congenic marker (002014, The Jackson Laboratory) for the tumor orthotopic models or with the CD45.2 congenic marker for the infiltration experiments. Experiments were performed in accordance with institutional and national guidelines and approved by the Experimental Animal Committee at the NKI and by the regional animal ethics committee of Northern Stockholm, Sweden under Ethical Permit number 5261-2020. All animals were housed in individually ventilated cage systems under specific-pathogen-free conditions. Both male and female mice were used at 8–12 weeks of age. Replicates of each experiment are described in the figure legends. The mice were randomised before the injection of the treated OT-I cells. No blinding was performed. All samples that passed the technical requirements were included (e.g., enough cells for flow cytometry).

### Human and mouse T cell cultures and treatments

Peripheral blood mononuclear cells (PBMCs) were obtained from healthy donors from Cambridge Bioscience, National Health Service (NHS) Blood and Transplant (NHSBT: Addenbrooke’s Hospital, Cambridge, UK) or Sanquin (Amsterdam, NL). The study was performed according to the Declaration of Helsinki (seventh revision, 2013). Ethical approval was obtained from the East of England-Cambridge Central Research Ethics Committee (06/Q0108/281) and consent was obtained from all subjects. Written informed consent was obtained (Cambridge Bioscience, Cambridge, UK; NHSBT Cambridge, UK; Sanquin Research, Amsterdam, NL). Human CD8^+^ T cells were isolated either directly after blood donation (8–12 h after blood collection) or they were cryopreserved and used after cryopreservation. PBMCs were isolated through Ficoll-Paque PLUS density gradient separation (GE Healthcare). Cells were incubated in 21% oxygen, 5% carbon dioxide at 37°C.

Human T cell isolation was performed with MACS Miltenyi kits (Naive CD8^+^ T cells: 130-093-244 or Total CD8^+^ T cells: 130-096-495) following manufacturer’s instructions. CD8^+^ T cells were activated with aCD3/CD28 beads (1:1 beads-to-cell ratio) (11132D, Gibco). OE-S-2HG treatment and OE-R-2HG (H942595; H942596 Toronto Research Chemicals) started at day 0 and was at 0.4 mM concentration, otherwise stated. Every second day, fresh complete RPMI media (52400-025; Gibco) containing 10% FBS, 1% penicillin-streptomycin and IL-2 (30 U/ml) and the appropriate amount of OE-S-2HG, OE-R-2HG or vehicle was added. Cell number and viability were measured by ADAM-MC automated cell counter (NanoEnTek) or by CASY cell counter and analyser (BIOKE). Rate of cell division was calculated by dividing the cell number of day (X+2) by cell number of day X.

Mouse splenic OT-I CD8^+^ T cell isolation was performed with MACS Miltenyi kit (CD8^+^ T cells: 130-104-075) following manufacturer’s instructions. OT-I T cells were activated with either SIINFEKL (1000 ng/mL) or by co-culturing them with pre-seeded OVA-expressing MEC.B7.SigOVA cells.^47^ The day after activation, the OT-I T cells were collected, washed, and treated with OE-S-2HG, OE-R-2HG or vehicle or 6–7 days. Cells were maintained in fresh complete RPMI media (52400-025; Gibco) containing 10% FCS, 1% penicillin-streptomycin, 55 μM β-mercaptoethanol and IL-2 (30 U/ml). B16-F10-OVA cells were cultured in DMEM high glucose with pyruvate (11995065 Thermo Scientific) containing 0.75 mg/mL G418 sulfate (10131027, Thermo Scientific).

## Method Details

### Flow cytometry

Human CD8^+^ T cells FACS analysis was performed on day 12, otherwise stated. Mouse CD8^+^ T cells were analyzed at day 7 of culture and OT-I adoptively transferred T cells were analyzed after blood or organ/tumor harvesting. Cells were pelleted by centrifugation and stained with antibodies in FACS Buffer (5% FBS, 2 mM EDTA in PBS) at 4°C for 30–60 min. CCR7 staining was performed in media at 37°C. The stained cells were then washed with FACS buffer, pelleted, and re-suspended in 1x FACS-Fix (BD CellFIXTM) and kept at 4°C in the dark until processing. The samples were processed 2–3 days after fixation. For intracellular cytokine staining, cells were cultured in the presence of 1 μg/mL brefeldin A and monensin (00-4505-51, eBioscience) for 2 h and were then fixed and permeabilized with Cytofix/Cytoperm kit (BD Biosciences) according to manufacturer’s protocol. For intracellular transcription factor and proliferation staining the Foxp3/Transcription Factor Staining (eBioscience) was used. On the day of analysis, the cells were resuspended in FACS Buffer containing counting beads (CountBrightTM Absolute Counting Beads). The number of cells in each sample was calculated according to the manufacturer’s instructions. Emission spectra “spillover” was corrected by compensation using compensation beads (01-1111-41; OneComp eBeads) mixed with each fluorescent probe. Flow cytometers used: BD LSR-Fortessa, AttuneX (Invitrogen) and BD FACSymphony. The flow data were analyzed using FlowJo (BD Biosciences, version 10). The antibodies used can be found in the “Key resources table”.

### Western blots and 5hmC staining

For western blots, human naive CD8^+^ T cells were isolated from healthy donors and treated from day 0 to day 12 with OE-S-2HG (0.4 mM), OE-R-2HG (0.4 mM) or vehicle. The cells were activated with aCD3/CD28 beads for 4 days as explained above. At day 12, cells were collected, washed, lysed and the histones were extracted by using the Histone Extraction Kit (ab113476, Abcam) the manufacturer’s instructions. Protein quantification was performed with the BCA protein assay kit (ab207003, Abcam). Proteins were separated by SDS-PAGE and transferred to PVDF membranes. Membranes were then blocked in 5% milk prepared in PBS with 0.05% Tween 20, incubated with primary antibodies overnight at 4°C and HRP-conjugated secondary antibodies (HAF008 and HAF007, R&D) for 1 h at room temperature the next day. Following ECL exposure (GERPN2106, Sigma), membranes were imaged using an iBrightCL1000 (Thermo Fisher). The primary antibodies used can be found in the “Key resources table”.

For 5hmC staining, naive CD8^+^ T cells were isolated from healthy donors and treated from day 0 to day 7 with OE-S-2HG (0.4 mM), OE-R-2HG (0.4 mM) or vehicle. The cells were activated with aCD3/CD28 beads for 4 days as explained above. At day 7, cells were collected, washed, and stained with Live/Dead (L34963, Invitrogen) and anti-human CD8a (53-6.7, Biolegend). The cells were then fixed (30 min at room temperature in the dark) and permeabilized (30 min at room temperature in the dark) (True-Nuclear Transcription Factor kit; Biolegend). After fixation and permeabilization, the cells were incubated with 4M HCL for 10 min at room temperature. The cells were then thoroughly washed and incubated in blocking buffer (0.1% PBS-Triton, 5% FCS) for 30 min at 4°C. The cells were then incubated either with primary anti-5hmC (10013602; Active Motif) or with isotype control antibody overnight at 4°C and the day after with secondary antibody for 1 h at room temperature. Flow cytometry was then performed as explained above.

### RNA-Seq analysis and GSEA

Naive CD8 T cells activated and treated with OE-S-2HG (0.4 mM), OE-R-2HG (0.4 mM) or vehicle and cells were collected on day 5 or day 12. The cells were lysed on RLT Buffer containing (1:100) β-Mercaptoethanol. RNA-Seq libraries were prepared and sequenced by Active Motif (USA) on Illumina NextSeq 500 for 42 cycles (42nt paried-end reads). Reads were quasi-mapped and quantified using Salmon (version 1.8),^60^ onto the transcriptome (ENSEMBL version 104).^61^ DESeq2 was used for differential expression analysis (cut-off: adjusted *p value* < 0.05 and absolute LFC>0.5). For GSEA analysis, we used counts outputted by STAR after mapping on the genome hg38 (ENSEMBL). GSEA analysis was performed with default setting to determine gene-set enrichment based on biological knowledge (e.g., genes sharing the same GO category). GSEAPreranked tool developed by Broad Institute: (http://software.broadinstitute.org/gsea/index.jsp).

The OE-R-2HG upregulated significant genes were used for enrichment analysis with the EnrichR tool^62^ and the ENCODE transcription factor ChIP-Seq library. The Human Protein Atlas (proteinatlas.org) and Monaco^63^ dataset was used to check the expression of specific genes in different immune cell types.

### *In vitro* enzymatic activity assays

For enzymatic activity of the KDM4C enzyme, the human KDM4C enzyme (8 nM) was incubated with the substrate of H3(1–21) lysine 9 tri-methylated biotinylated peptide (30 nM), in the presence of αKG (10 μM), ammonium iron(II) sulfate hexahydrate (5 μM), assay buffer (50 mM HEPES pH 7.0, 0.01% Tween 20, 1mM ascorbic acid and 0.01% BSA). S-2HG (Sigma) and R-2HG (Sigma) were used in 3-fold serial dilution and the maximum concentration used was 5 mM. The pre-incubation time of the inhibitors with the KDM4C mixture was 30 min at room temperature. The reaction step was at room temperature for 210 min. The product was detected by using an anti-histone H3K9 Me2-Eu(K) antibody and XL665-conjugated Streptavidin. The detection of the homogeneous time resolved fluorescence (HTRF) signal was proportional to the concentration of demethylated H3(1–21) peptide. The assays were performed in technical duplicates in a 384-well plate and repeated 2–3 independent times. The inhibitor analysis was performed within the linear range of catalysis.

For enzymatic activity of the TET2 enzyme, the human TET2 enzyme (2 nM) was incubated with the substrate of ssDNA (ssBiotin 26nt Me-C Oligo 30 nM), in the presence of αKG (115 μM), ammonium iron(II) sulfate hexahydrate (10 μM), in assay buffer (50 mM HEPES pH 7.0, 100 mM NaCl, 0.01% Pluronic F-127, 1mM TCEP, 2mM ascorbic acid, 0.2 mg/mL BSA and 1000U/ml Catalase). S-2HG (Sigma) and R-2HG (Sigma) were used in 3-fold serial dilution and the maximum concentration used was 5 mM. The pre-in-cubation time of the inhibitors with the TET2 mixture was 30 min at room temperature. The reaction step was at room temperature for 90 min. The product was detected by using an anti-5-Hydroxymethylcytosine antibody (5 nM), Eu-Protein A (5 nM), Streptavidin-Alexa Fluor 647 (6.25 nM) and 10 mM EDTA. For the standard curve, the ssBiotin 26nt HydMe-C Oligo was used. The assays were performed in technical duplicates in a 384-well plate and repeated 2–3 independent times. The inhibitor analysis was performed within the linear range of catalysis.

The percentage of inhibition was calculated with the following formula: Inhibition%=(1-(signal value per well-Average Low control)/ (Average High control-Average Low control))*100. The data were fitted by Prism Graphpad with four parameters equation via “log(inhibitor) vs. response – Variable slope” model.

### Quantification of R- and S-2HG by LC-MS

Pelleted T-cells were extracted in 200 μL of ice-cold 80% (v/v) methanol in water were added to the cell pellets. 2 μL of a 1 mM (RS)-2HG-d_3_ deuterated internal standard (IS) was added at this step to correct for possible losses during the sample preparation and for matrix effects, as well as aiding in the correct annotation of 2HG enantiomers. Samples were sonicated for 1 min and kept at -20°C for 30 min to allow for protein precipitation. After centrifugation at 18000xg and 4°C for 10 min supernatants were transferred to new 1.5 mL Eppendorf tubes. Extraction was repeated with 100 μL 80% (v/v) methanol and supernatants were combined. Samples were dried using a SpeedVac (Eppendorf) until fully dry.^64^

Derivatization of the dried pellets took place by adding 50 μL of 50 mg/mL L-DATAN in 4:1 (v/v) acetonitrile:acetic acid to form the corresponding 2HG-DATAN diastereomers that can be separated under achiral chromatographic conditions. To favor the derivatization, samples were placed in a heat block for 2 h at 80°C after which they were briefly spun down and fully dried under nitrogen gas stream. After reconstitution with 100 μL 4:1 (v/v) water:acetic acid the samples were transferred to glass inserts, placed in LC vials and were subjected to LC-MS analysis. Similarly, a calibration line was constructed using R- and S-2HG standard solutions having a final concentration from 0.5 to 200 μM of each enantiomer. Each calibration line solution was spiked with the same amount of IS than for the samples and were likewise derivatized and analyzed.

All the samples and calibration line solutions were analyzed by liquid chromatography coupled to mass spectrometry (LC-MS) using so-called enhanced in-source fragmentation to enable MS fragmentation.^64^ The analyses were conducted in an ultra-high-performance LC Dionex UltiMate 3000 UHPLC (Thermo Fisher) system coupled to a Bruker Impact II Q-TOF system (Bruker) provided with a Develosil Aqua C30 column (3 μm; 2 mm inner diameter (i.d.) ×150 mm length) and a Phenomenex C8 2.1 mm i.d. precolumn. LC eluents were pumped at a 0.4 mL/min flow rate having as mobile phase A 125 mg/L of ammonium formate in water (pH 3.6) and as mobile phase B 95% (v/v) of acetonitrile in water. The chromatographic gradient composition was 0% B at 0.0 min, 36% B at 5.0 min, 100% B at 6.0 min, 100% B at 8.0 min, 0% B at 8.4 min and 0% B at 13.0 min. S- and R-2HG as well as their respective enantiomeric deuterated versions (IS) eluted at 3.7 and 3.9 min, respectively, and were monitored using the ions *m/z* 363.0570 + 147.0270 (for 2HG) and *m/z* 366.0745 + 150.0491 (for the deuterated 2HG IS).

### NMR-based metabolomics

Sample preparation for the quantification of metabolites by nuclear magnetic resonance (NMR) spectroscopy cells were washed once with PBS, centrifuged and quenched in liquid nitrogen.^65^ Polar metabolites were extracted with 200 μL of an ice-cold solution of 67.5:7.5:25 (v/v/v) of methanol/chloroform/water at -20°C. Samples were sonicated for 1 min, were kept on dry-ice for 30 min and were further centrifuged at 18,000xg for 15 min at 4°C. The supernatant was collected and fully dried under nitrogen gas stream. Samples were reconstituted in 220 μL of a 50 mM phosphate buffer (pH 7.4) solution in deuterated water containing 0.05 mM trimethylsilyl propionic-d_4_-sodium salt (TSP-*d*_*4*_, Cambridge Isotope Laboratories, Inc.) as internal standard used for NMR referencing and quantification. One NMR experiment (pulse sequence: *noesygppr1d*; Bruker Biospin Ltd) was collected for each sample in a 14.1 T (600 MHz for ^1^H) Bruker Avance *Neo* NMR. All recorded NMR spectra were imported in Chenomx NMR suite 9.0 (Chenomx NMR suite, v9.0, Edmonton, AB, Canada) for the quantification of metabolites. Quantitative data (pmoles) was then normalized to the number of cells of each sample.

### Simulations

#### System setup

The atomic structure of the KDM4C protein (PDB id 4XDO; resolution 1.97 Å)^66^ in complex with αKG (KDM4C:αKG: Fe(II)) was used as a starting point for Molecular Dynamics (MD) simulations. Three systems were prepared: KDM4C:αKG:Fe(II), KDM4C:2HG-(*S*):Fe(II) and KDM4C:2HG-(*R*):Fe(II). Calculations were done with NAMD 2.9,^55^ using the CHARMM 36 protein force-field^49^ together with the TIP3P water model.^67^ 2OG, 2HG-(*S*), and 2HG-(*R*) parameters were obtained in CHARMM from Paramchem.^68^ Fe(II) Lennard Jones parameters were taken from the CHARMM force field. Default ionization states were used for the protein on the basis of PropKa calculations.^69^

#### Simulation details

The particle mesh Ewald (PME) algorithm was used for the evaluation of electrostatic interactions beyond 12 Å, with a PME grid spacing of 1 Å, and NAMD defaults for spline and κ values.^58^ A cutoff at 12 Å was applied to nonbonded forces. Both electrostatics and van der Waals forces were smoothly switched off between the cutfoff distance of 12 Å and the switching distance of 10 Å using the default NAMD switching function. A Verlet neighbor list^70^ with pairlist distance of 14 Å was used only to evaluate nonbonded neighboring forces within the pairlist distance. The lengths of covalent bonds involving hydrogen atoms were constrained by the SETTLE algorithm^59^ to be able to use a 2 fs timestep. The multi time step algorithm Verlet-I/r-RESPA^54^ was used to integrate the equations of motion. Nonbonded short-range forces were computed for each time step, while long-range electrostatic forces were updated every 2 timesteps. The pressure was kept at 1.026 bar (1 atm) by the Nosé -Hoover Langevin piston,^56,57^ with a damping time constant of 50 fs and a period of 100 fs. The temperature was maintained at 300 K by coupling the system to a Langevin thermostat,^51^ with a damping coefficient of 5 ps^−1^. A 150 mM background ionic concentration of NaCl was utilized to achieve system neutrality. The total system size was 94,691 atoms, comprised of 8,264 waters, 84 Na^+^ ions and 88 Cl^-^ ions.

After 1,000 steps of Conjugate-Gradient minimization^50^ with restraints on the protein and co-factor, and 10 ns of simulation with backbone restraints and restraints on the co-factor, a 50 ns production run in the NPT ensemble was carried out for each system.

### Animal studies

For infiltration experiments, 8-12-week-old male and female C57BL/6J/Ly5.1/Ly5.2 mice were injected subcutaneously with 1 x 10^6^ B16-OVA cells and conditioned 11 days later with peritoneal injection of 300 mg/kg cyclophosphamide (Sigma, #C0768). On day 14, 1 x 10^6^ OT-I Ly5.2 CD8^+^ T cells were peritoneally injected (the OT-I cells were previously activated and treated with 0.4 mM OE-S-2HG, OE-R-2HG or vehicle *in vitro* for 7 days). Animals were assigned randomly to each experimental group. On day 19, tumors, spleens, and lymph nodes (draining and non-draining) were harvested. The excised tumors were cut into small pieces and digested with 100 μg/mL DNase I and 200 U/ml Collagenase at 37°C for 30 min. The spleens and lymph nodes were smashed over a 40 μm filter. Cells were counted, and where indicated, they were re-stimulated for 4 h with 100 nM OVA_257–264_ peptide and brefeldin A and monensin were added for the last 2 h of activation. The tumor single-cell suspensions were stained with fluorochrome-labelled antibodies and analyzed by flow cytometry.

For tumor growth experiments, 8-15-week-old female C57BL/6J/Ly5.2 were inoculated subcutaneously with 0.5 x 10^6^ B16-F10-OVA and conditioned 4 days later with peritoneal injection of 300 mg/kg cyclophosphamide. On day 7, 0.5 x 10^6^ OT-I Ly5.1 CD8^+^ T cells were peritoneally injected (the OT-I cells were previously activated and treated with 0.4 mM OE-S-2HG, OE-R-2HG or vehicle *in vitro* for 7 days). Animals were assigned randomly to each experimental group. Tumor volume was measured every 2–3 days with electronic calipers until day 60. Peripheral blood was collected from the tail vein at days 14 and 21 and analyzed by flow cytometry. Tumor volume was calculated using the formula a×b×b/2 where a is the length and b is the width of the tumor. Mice were sacrificed when the tumors reached a size of 500 mm^3^.

## Quantification and Statistical Analysis

Results are shown as mean ± SEM or mean ± SD as stated in figure legends. Statistical analysis was performed with Prism-9 software (Graph-Pad). Statistical significance was set at p < 0.05 and the statistical tests used are stated in figure legends.

### BioRender images

The graphical abstract and the panels Figures 4A and S3B were created with BioRender.com with agreement numbers GF25OEXDR7, LK25NAR4UU and UL25NAS2IF respectively.

## Supplementary Material

Supplemental Information

## Data Availability

The RNA-Seq data generated in this study are publicly available in Gene Expression Omnibus (GEO): GSE212738. Some original and all analyzed data are publicly available in Mendeley Data: https://doi.org/10.17632/gj337xjxk6.1. Scripts/codes used in the paper are available at: https://github.com/BenNicolet/RNA-Seq-2HG-Foskolou-et-al-2023; https://github.com/chrisjorg/MDscripts-RS Any additional information required to reanalyze the data reported in this work paper is available from the lead contact upon request.

## In brief

Foskolou et al. show that the enantiomeric forms S-2HG and R-2HG have distinct functions in CD8^+^ T proliferation, differentiation, and function. Treatment of CD8^+^ T cells with exogenous cell-permeable S-2HG, but not R-2HG, increased CD8^+^ T cell fitness *in vivo* and enhanced anti-tumor activity.

## Highlights

- Octyl-ester S-2HG and R-2HG differentially modulate CD8^+^ T cell differentiation
- S-2HG and R-2HG have different inhibitory potencies against aKG-dependent enzymes
- CD8^+^ T cells pre-treated with octyl-ester S-2HG have enhanced anti-tumor activity
