# Supplementary material for: The two enantiomers of 2-hydroxyglutarate differentially regulate cytotoxic T cell function
Source: Cell Rep. Author manuscript; Available in PMC 2025 Sep 15. (PMC7618115; doi:10.1016/j.celrep.2023.113013)
Supplement: Supplemental Information [file EMS207964-supplement-Supplemental_Information.zip › 1-s2.0-S2211124723010240-mmc1.pdf]

Cell Reports, Volume *42*

Supplemental information

The two enantiomers of 2-hydroxyglutarate differentially regulate cytotoxic T cell function

Iosifina P. Foskolou, Pedro P. Cunha, Elena Sánchez-López, Eleanor A. Minogue, Benoît P. Nicolet, Aurélie Guislain, Christian Jorgensen, Sarantos Kostidis, Nordin D. Zandhuis, Laura Barbieri, David Bargiela, Demitris Nathanael, Petros A. Tyrakis, Asis Palazon, Martin Giera, Monika C. Wolkers, and Randall S. Johnson

Supp. Figure 1

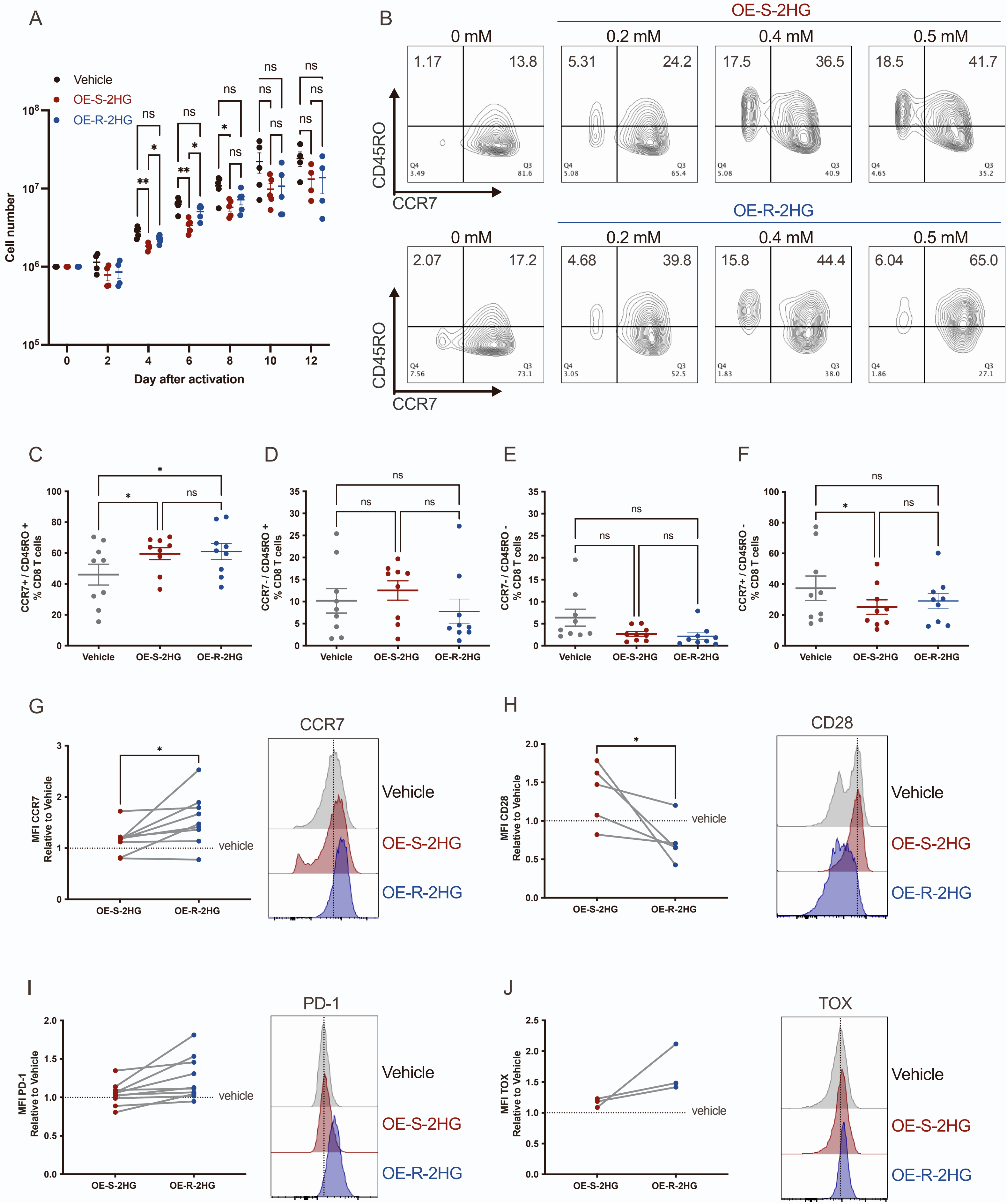

Supp. Figure 1: Expression of surface markers in human CD8+ T cells treated with OE-S-2HG or OE-R-2HG. (Related to Figure 1)

(A) Cell number of CD8+ T cells treated with OE-S-2HG (0.4 mM), OE-R-2HG (0.4 mM) or vehicle (H_2O) for the indicated days as determined by an automated cell counter. Data are represented in log10 as mean ± SEM. Mixed-effects analysis with Tukey's multiple comparisons test was used. **(B)** Flow cytometry plots of CD8+ T cells showing surface expression of CCR7 and CD45RO. Cells were treated with vehicle (H_2O) or increasing concentrations of OE-S-2HG or OE-R-2HG and analysed at day 12 by flow cytometry. Representative plots of n= 3 is shown. **(C-F)** Cells were treated with OE-S-2HG (0.4 mM), OE-R-2HG (0.4 mM) or vehicle (H_2O) and the proportion of (C) CCR7+/CD45RO+; (D) CCR7-/CD45RO+; (E) CCR7-/CD45RO-; (F) CCR7+/CD45RO- cells is shown (%CD8+ T cells). Cells were analysed at day 12/13 by flow cytometry. Each data point represents a donor (n= 9; from 5 independent experiments). Data are represented as mean ± SEM. RM one-way ANOVA with Tukey's multiple comparisons test was used. **(G)** Fold change of median fluorescence intensity (MFI) of CCR7 for CD8+ T cells treated with OE-S-2HG (0.4 mM) or OE-R-2HG (0.4 mM) relative to vehicle (H_2O) on the left, and representative histogram flow cytometry plots on the right. Cells were analysed at day 12. Each data point represents a donor (n= 9; from 6 independent experiments). Unpaired two-tailed Student t test was used. **(H)** Fold change of median fluorescence intensity (MFI) of CD28 for CD8+ T cells treated with OE-S-2HG (0.4 mM) or OE-R-2HG (0.4 mM) relative to vehicle (H_2O) on the left, and representative histogram flow cytometry plots on the right. Cells were analysed at day 12/13. Each data point represents a donor (n= 5; from 3 independent experiments). Unpaired two-tailed Student t test was used. **(I)** Fold change of median fluorescence intensity (MFI) of PD-1 for CD8+ T cells treated with OE-S-2HG (0.4 mM) or OE-R-2HG (0.4 mM) relative to vehicle (H_2O) on the left, and representative histogram flow cytometry plots on the right. Cells were analysed at day 12. Each data point represents a donor (n= 10; from 6 independent experiments). Unpaired two-tailed Student t test was used. **(J)** Fold change of median fluorescence intensity (MFI) of TOX for CD8+ T cells treated with OE-S-2HG (0.4 mM) or OE-R-2HG (0.4 mM) relative to vehicle (H_2O) on the left, and representative histogram flow cytometry plots on the right. Cells were analysed at day 15. Each data point represents a donor (n= 3). Unpaired two-tailed Student t test was used. For all panels naïve CD8+ T cells were isolated and activated with CD3/CD28 beads and cultured with IL2 (30

U/mL) in the presence of OE-S-2HG (0.4 mM), OE-R-2HG (0.4 mM) or vehicle (H_2O) from day 0 to 12, unless otherwise stated.

For all panels: *P ≤ .05; **P ≤ .01; ***P ≤ .001; ****P ≤ .0001.

Supp. Figure 2

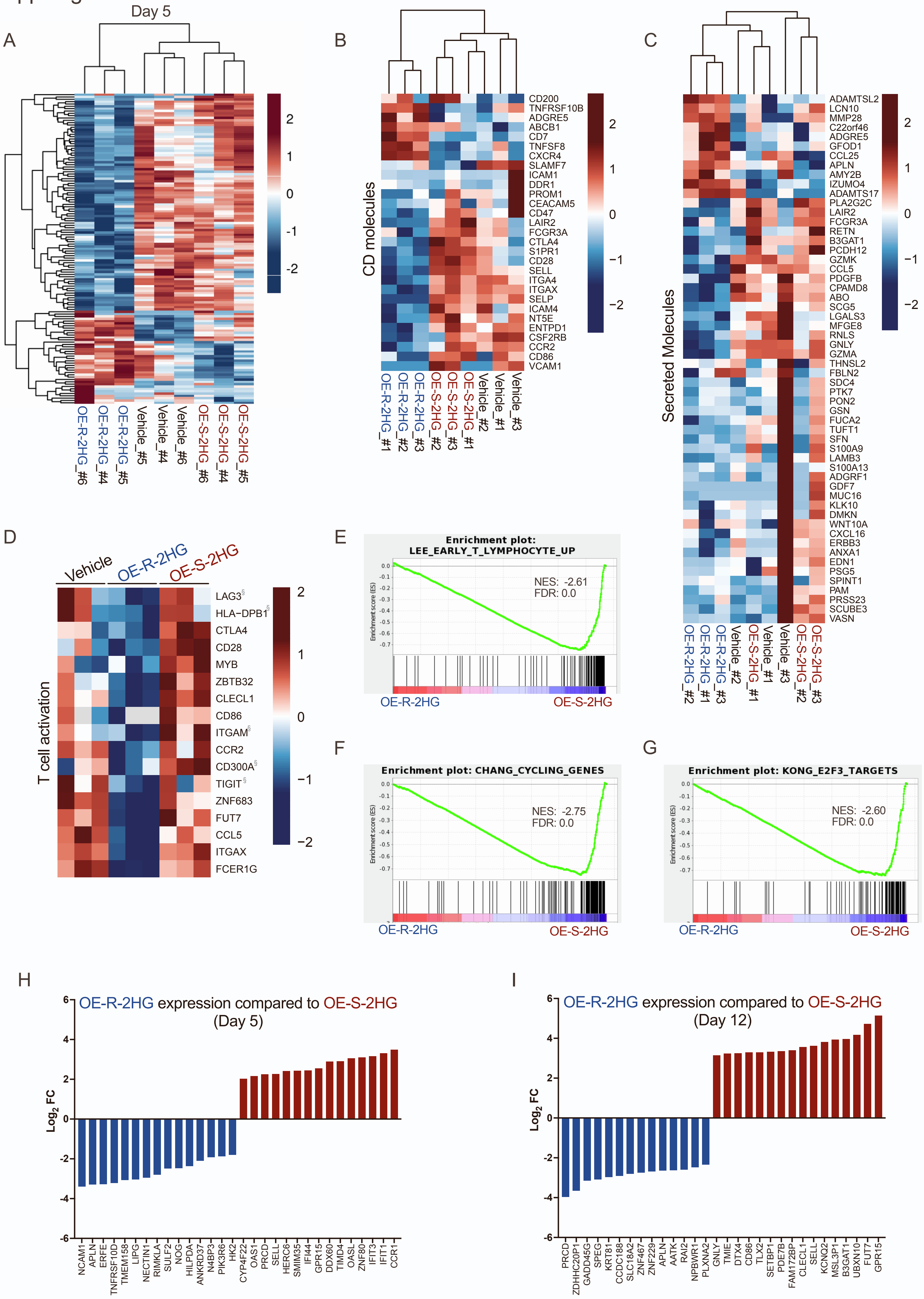

Supp. Figure 2: RNA-Seq analysis of OE-S-2HG and OE-R-2HG treated human CD8+ T cells. (Related to Figure 2)

Naïve CD8+ T cells were isolated from 6 individual donors over 3 independent experiments, activated and treated with OE-S-2HG (0.4 mM), OE-R-2HG (0.4 mM) or vehicle (H_2O). The cells were collected either on day 5 (3 donors) or on day 12 (3 donors) and RNA-Seq analysis followed. **(A)** Heatmap of hierarchically clustered genes in CD8+ T cells treated with OE-S-2HG (0.4 mM), OE-R-2HG (0.4 mM) or vehicle (H_2O) at day 5 of culture. **(B-C)** Heatmaps of hierarchically clustered genes in CD8+ T cells treated with OE-S-2HG (0.4 mM), OE-R-2HG (0.4 mM) or vehicle (H_2O) at day 12 of culture. Statistically significant differentially expressed hits of (B) CD molecules and (C) secreted molecules are shown. **(D)** Heatmap of standardized gene expression (Z score) in treated CD8+ T cells of genes involved in T cell activation. The gene set was obtained from ToppGene. Red and blue colours indicate increased and decreased expression respectively. Genes marked with '§' were not statistically significant hits. Samples from day 12 of treatment are shown. **(E-G)** Gene set enrichment analysis (GSEA) of treated CD8+ T cells at day 12 of culture for (E) Lee early T lymphocytes up; (F) Chang cycling genes and (G) Kong E2F3 targets. Net enrichment score (NES) values and false discovery rate (FDR) are shown. **(H-I)** Plots show the top 30 most differentially expressed genes for (H) day 5 and (I) day 12 of CD8+ T cells treated with OE-S-2HG (0.4 mM) or OE-R-2HG (0.4 mM).

Supp. Figure 3

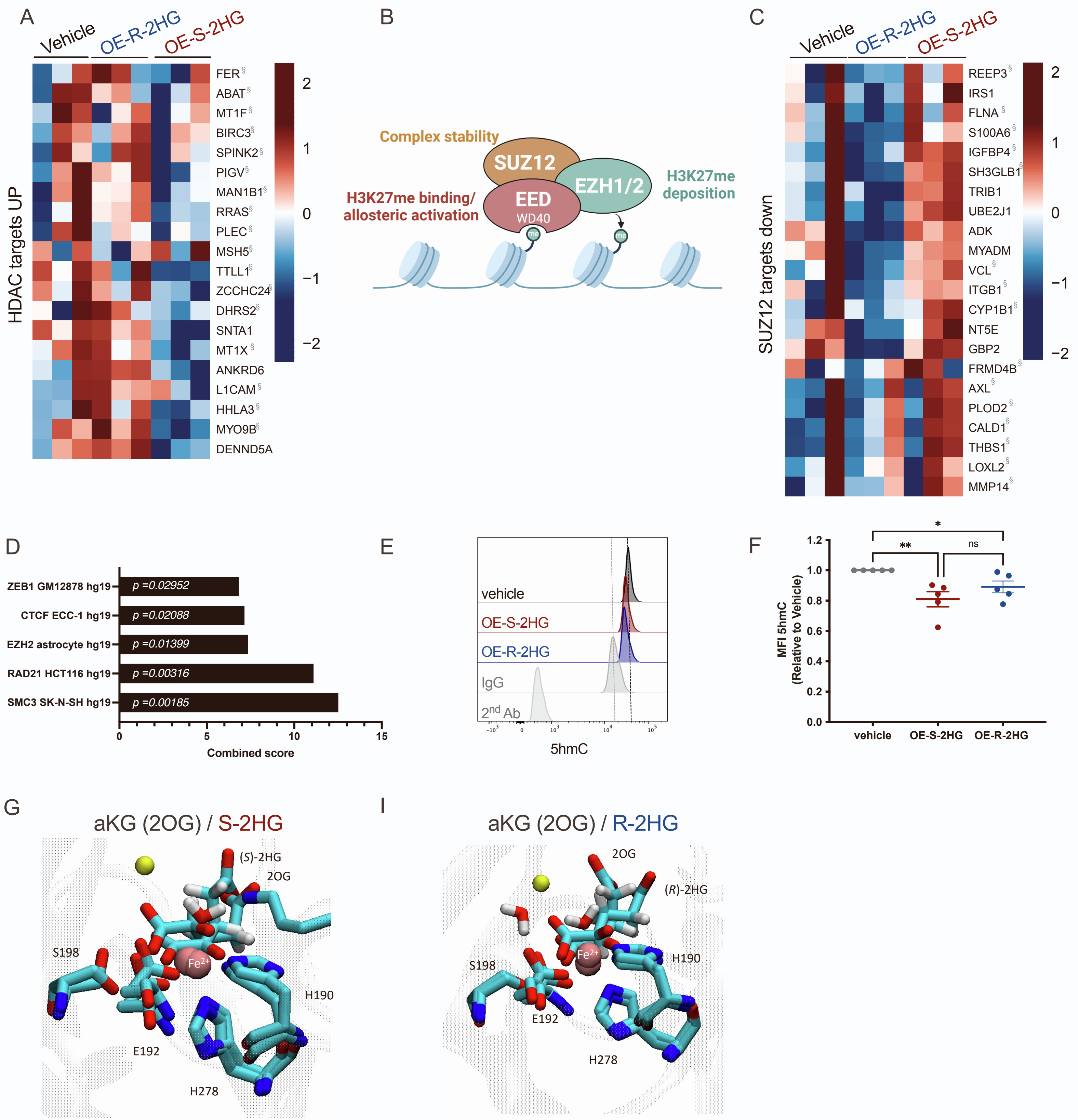

Supp. Figure 3: Mechanistic insight of OE-S-2HG and OE-R-2HG treatment in CD8+ T cells. (Related to Figure 3)

(A) RNA-Seq was performed as described in figure 2. Heatmap of standardized gene expression (Z score) in treated CD8+ T cells for HDAC gene targets (upregulated). The gene set was obtained from ToppGene. Red and blue colours indicate increased and decreased expression respectively. Genes marked with '§' were not statistically significant hits. Samples from day 12 of treatment are shown. **(B)** Schematic representation of the PRC2 complex is shown. **(C)** RNA-Seq was performed as described in figure 2. Heatmap of standardized gene expression (Z score) in treated CD8+ T cells for SUZ12 targets down (downregulated). The gene set was obtained from ToppGene. Red and blue colours indicate increased and decreased expression respectively. Genes marked with '§' were not statistically significant hits. Samples from day 12 of treatment are shown. **(D)** OE-R-2HG upregulated significant hits were used for enrichment analysis with the EnrichR tool [52] using the the ENCODE TF ChIP-seq 2015 data set. The combined scores of significant targets are shown. **(E-F)** Naïve CD8+ T cells were activated and treated with vehicle (H_2O), OE-S-2HG (0.4 mM) or OE-R-2HG (0.4 mM) and 7 days after the intracellular levels of 5hmC were measured by flow cytometry. (E) Representative histogram flow cytometry plots for 5hmC (IgG: isotype control, 2nd Ab: secondary antibody staining only). (F) Fold change of median fluorescence intensity (MFI) of 5hmC for CD8+ T cells relative to vehicle. Each data point represents a donor (n= 5; from 2 independent experiments). Data are represented as mean ± SEM. Unpaired two-tailed Student t test was used between treatments. **(G)** Overlay images of the catalytic site of KDM4C protein (PDB id 4XDO; resolution 1.97 Å) in complex with αKG (KDM4C:2OG:Fe(II)) and S-2HG (KDM4C:2HG-(S):Fe(II)). **(I)** Overlay images of the catalytic site of KDM4C protein (PDB id 4XDO; resolution 1.97 Å) in complex with αKG (KDM4C:2OG:Fe(II)) and R-2HG (KDM4C:2HG-(R):Fe(II)). For panel E naïve CD8+ T cells were isolated and activated with CD3/CD28 beads and cultured with IL2 (30 U/mL) in the presence of OE-S-2HG (0.4 mM), OE-R-2HG (0.4 mM) or vehicle (H_2O) from day 0 to 7.

For all panels: *P ≤ .05; **P ≤ .01; ***P ≤ .001; ****P ≤ .0001.

Supp. Figure 4

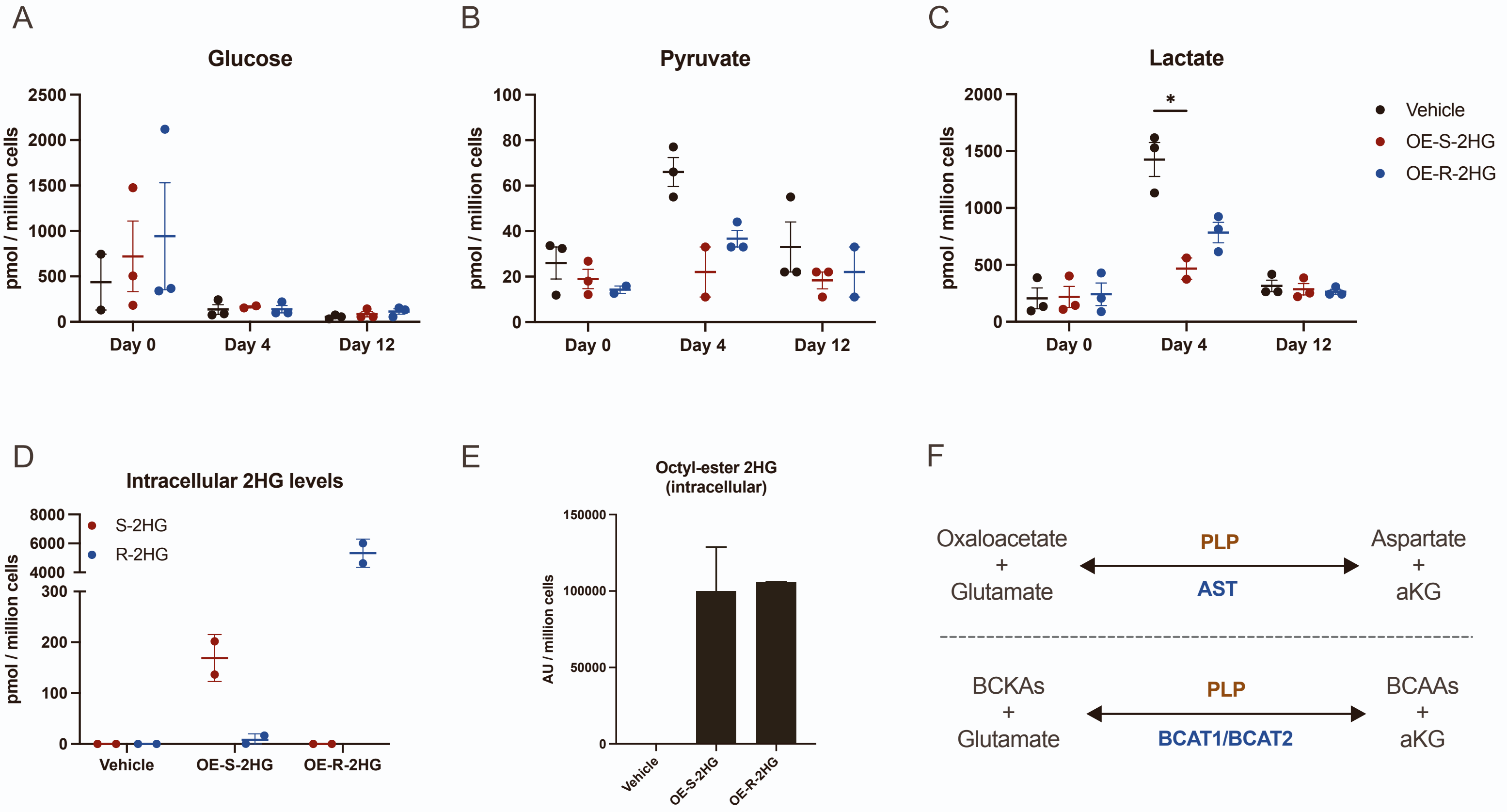

Supp. Figure 4: Metabolic changes in OE-S-2HG and OE-R-2HG treated total CD8+ T cells early and late after activation. (Related to Figure 4)

(A-C) Total CD8+ T cells were isolated from 3 individual donors, activated, and treated every one to two days with OE-S-2HG (0.4 mM), OE-R-2HG (0.4 mM) or vehicle (H_2O). The cells were collected either before activation or after activation on day 4 and day 12 and NMR-based metabolomics followed. The levels of (A) glucose, (B) pyruvate and (C) lactate were measured. Data are represented as mean ± SEM. Two-way ANOVA with mixed- effects analysis and Tukey's multiple comparisons tests were used. **(D-E)** Total CD8+ T cells were activated for 4 days and treated with OE-S-2HG (0.4 mM), OE-R-2HG (0.4 mM) or vehicle and the amount of (D) intracellular S-2HG and R-2HG (data are represented as mean ± SD; n= 2 donors) or (E) octyl-ester 2HG was measured by LC-MS 20hrs after treatment (data are represented as mean ± SEM; n= 2 donors). The amount is represented either as pmol or arbitrary units (AU) per million cells. **(F)** Schematic representation of some αKG-dependent transaminases. Different transaminases recognise different amino acids, and they all use αKG and glutamate as a α-keto acid / α-amino acid pair. The upper reaction shows the production of aspartate by its cognate α-keto acid oxaloacetate, and the lower reaction shows the production of altered branched-chain amino acids (BCAAs) to the respective branched-chain α-keto acids (BCKAs). Aspartate transaminase (AST); BCAA aminotransferase 1/2 (BCAT1/2); Pyridoxal phosphate (PLP).

Panel C: *P ≤ .05

Supp. Figure 5

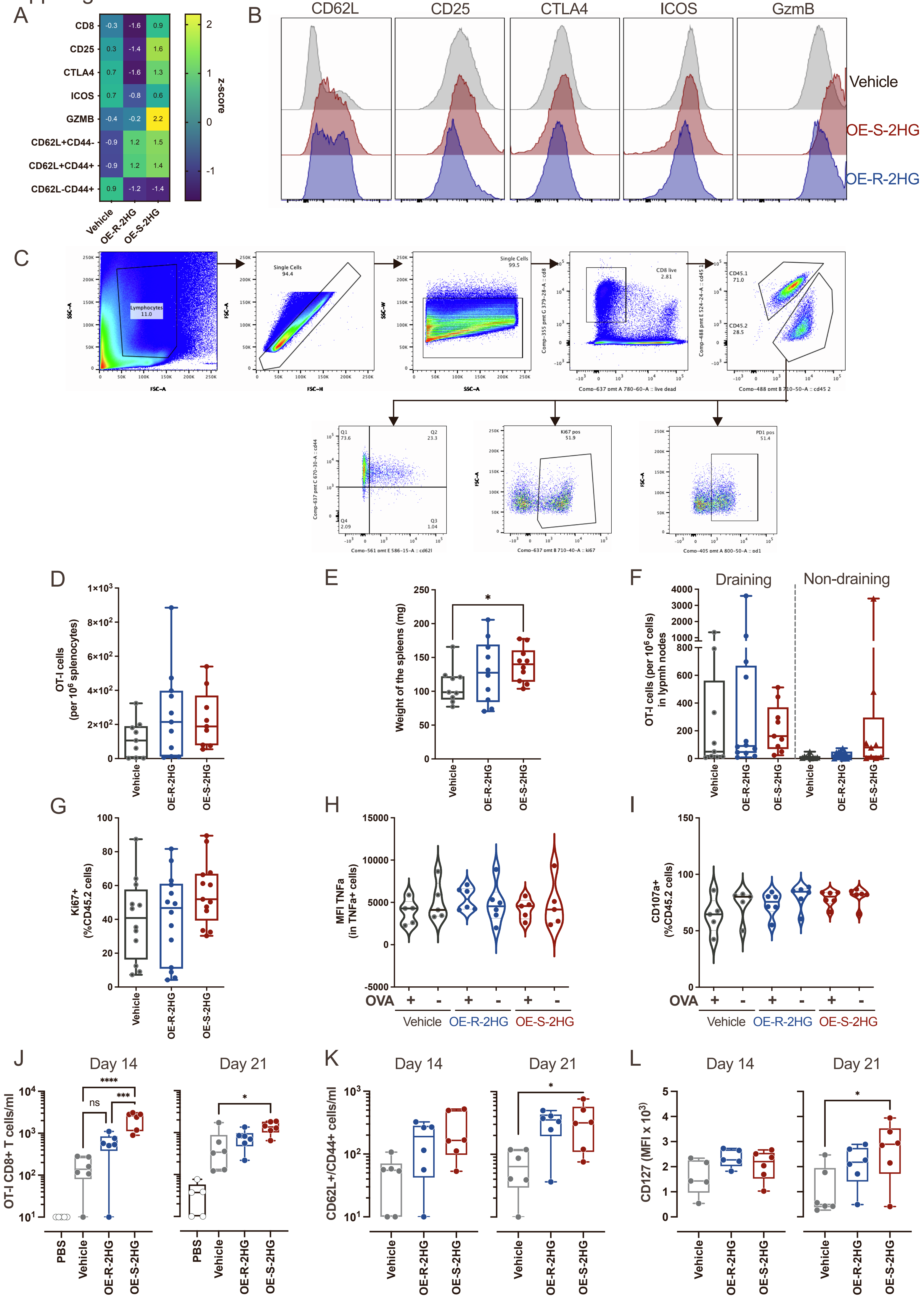

Supp. Figure 5: OE-S-2HG and OE-R-2HG treated mouse CD8+ T cells in adoptive cell transfer model. (Related to Figure 5)

(A) Heatmap of standardized expression (Z score) for specific markers or percentage of specific populations in OT-I CD8+ T cells treated with vehicle (H_2O), OE-R-2HG (0.4 mM) or OE-S-2HG (0.4 mM) for 7 days *in vitro*. **(B)** Representative histogram flow cytometry plots of the markers tested in (A). **(C)** Gating strategy of the OT-I tumour infiltrated lymphocytes. The host CD8+ T cells were CD45.1+CD45.2+ positive and the adoptive transferred OT-I cells were CD45.2+. **(D)** Number of adoptively transferred OT-I cells per million splenocytes. Cell number was defined by counting beads. Median and min to max with all points shown (n= 9-11 mice per condition, two independent experiments; non-parametric Kruskal-Wallis with Dunn's multiple comparisons test). **(E)** Weights of spleens from mice treated with OE-S-2HG, OE-R-2HG or vehicle OT-I cells. Median and min to max with all points shown (n= 9-10 mice per condition, two independent experiments; unpaired two-tailed Student t test). **(F)** Number of adoptively transferred OT-I cells in draining and non-draining lymph nodes. Cell number was defined by counting beads and the amount of the OT-I cells per million cells in lymph nodes was calculated. Median and min to max with all points shown (n= 9-11 mice per condition, two independent experiments; non-parametric Kruskal-Wallis with Dunn's multiple comparisons test was performed for each draining and non-draining lymph nodes). **(G)** Frequency of adoptively transferred OT-I cells (CD45.2+) expressing Ki67+ infiltrated in the tumours. Median and min to max with all points shown (n= 12-14 mice per condition, three independent experiments; ordinary one-way ANOVA with Holm-Sidak's multiple comparisons test). **(H-I)** Restimulation of OT-I tumour infiltrated lymphocytes *in vitro* with $OVA_{257\text{-}264}$ (100 nM) peptide for 4 hrs. Brefaldin A and monensin were added at the last 2 hrs before flow cytometry analysis. (H) TNFα median fluorescent intensity (MFI) of adoptively transferred OT-I cells (CD45.2+, TNFα+) cells with (+OVA) or without (-OVA) restimulation. (I) Frequency of adoptively transferred OT-I cells (CD45.2+) expressing CD107a with (+OVA) or without (-OVA) restimulation. Violin plots with median and all points is shown. Representative of n= 2-3 independent experiments is shown (cumulative data from n=3 is deposited in Mendeley Data). One-way ANOVA with Tukey's multiple comparisons was used between treatments and paired two-tailed Student t test was used for each treatment with (+OVA) compared to without (-OVA) restimulation. **(J)** Frequency of adoptively transferred OT-I cells per millilitre of peripheral blood on day

14 (left) and day 21 (right). Median and min to max with all points shown of n= 5-6 mice per condition. Ordinary one-way ANOVA with Tukey's multiple comparisons was used. **(K)** Frequency of adoptively transferred OT-I cells positive for CD62L+/CD44+ markers per millilitre of peripheral blood on day 14 (left) and day 21 (right). Median and min to max with all points shown of n= 5-6 mice per condition. Ordinary one-way ANOVA with Tukey's multiple comparisons was used. **(L)** CD127 median fluorescent intensity (MFI) of adoptively transferred OT-I cells circulating in peripheral blood on day 14 (left) and day 21 (right). Median and min to max with all points shown of n= 5-6 mice per condition. Ordinary one-way ANOVA with Tukey's multiple comparisons was used.

For all panels: *P ≤ .05; **P ≤ .01; ***P ≤ .001; ****P ≤ .0001.

Table S1

	T_N	T_{CM}	T_{EM}	T_{EMRA}
**SELL**	2432	1596	413	425
**CD28**	99	199	95	12
**GPR15**	1	49	14	5
**NT5E**	126	71	12	9
**CLEC2D**	452	408	524	482
**FUT7**	3	18	15	6
**ZNF69**	23	14	3	4
**ZNF683**	3×10^{-1}	91	339	502
**TIAM1**	10	54	11	3
**MGAT3**	0	1	2	5
**SLC16A10**	9	2	1	1
**FKBP5**	456	274	203	131
**IKZF3**	59	107	110	140
**PBX4**	5	6	4	4

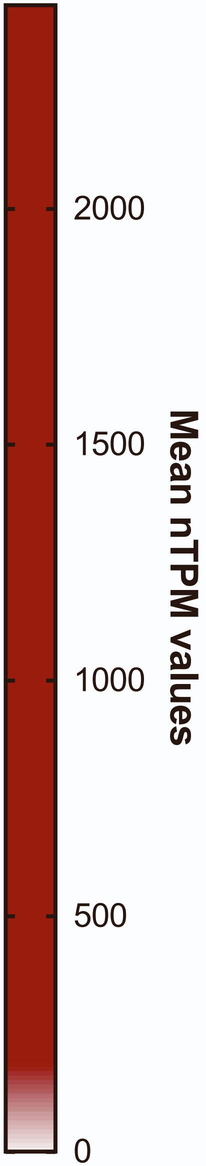

Table S1: Expression levels of genes upregulated in OE-S-2HG or OE-R-2HG samples in specific CD8+ T cell subsets. (Related to Figure 2)

Gene expression of specific targets is shown for naïve CD8+ T cells (T_N), central memory CD8+ T cells (T_{CM}), effector memory CD8+ T cells (T_{EM}) and terminally differentiated effector memory CD8+ T cells (T_{EMRA}). The values of each target were taken by the Monaco dataset [53] through Human Protein Atlas. The data were normalised using trimmed mean of M values (TMM) to allow for between-sample comparisons. The resulting normalised transcript expression values, denoted nTPM, were calculated for each gene in every sample.
